# Advances in biomanufacturing and recent biological applications of the fundamental bacterial carotenoids: a comprehensive review

**DOI:** 10.1186/s12934-025-02847-1

**Published:** 2025-10-27

**Authors:** Khaled B. Al-Monofy, Mahmoud H. Farghali, Lamiaa A. Al-Madboly, Amal M. Abo Kamer, Ahmed A. Abdelaziz

**Affiliations:** Department of Microbiology and Immunology, Faculty of Pharmacy, Tanta University, Tanta, Egypt

**Keywords:** Natural pigments, Bacterial pigments, Carotenoids, Production optimization, Genetic engineering, Biological applications

## Abstract

Many research teams have prioritized the investigation of the biotechnological production of carotenoids in the last few decades due to their multipurpose application. In this review, the bioproduction of the fundamental bacterial carotenoids, including lycopene, β-carotene, β-cryptoxanthin, zeaxanthin, canthaxanthin, and astaxanthin, is discussed, along with detailed information on their biosynthesis, laboratory screening, physiochemical requirements, genetic engineering-based production development, and fermentation techniques. As well, this review emphasizes that the bacterial carotenoids are innovative therapeutic avenues with diverse biological applications, from food additives to anticancer agents. Moreover, the review discusses the limitations of the application of these carotenoids and the future perspectives. Ultimately, the review highlights the bacteria as powerful cell factories, potentially facilitating the commercial production of carotenoids in the future.

## Introduction

Pigments are substances that, via their absorption of light, give materials their distinctive colors [1]. Both natural and synthetic approaches can be used to create pigments [2]. When compared to their natural counterparts, the synthetic pigments are far more affordable, easily applied, available in a wide range of colors, and their production is reproducible. Despite the wide application of synthetic dyes, the usage of natural dyes has increased greatly due to synthetic dyes’ drawbacks [3]. Since most customers prefer natural products, natural pigments are more widely approved for use in food, supplements, feed additives, and natural colorants than chemically synthesized ones [4]. Furthermore, people generally have a negative view of synthetic items, and large dosages of synthetic pigments may have harmful health effects [4]. For instance, children’s behavioral problems have been linked to the use of artificial food coloring [5]. The food industry’s interest in natural colorants has actually increased as a result of these facts. Even though legislation that distinguishes between “natural” and “artificial” food coloring is not entirely clear in the US and the EU [5]. Between 2026 and 2033, the demand for natural pigments is expected to expand steadily at a 4.5% compound annual growth rate (CAGR) (https://www.marketresearchintellect.com/product/global-natural-pigment-market-size-and-forecast/). In contrast, the global synthetic dyes market is expected to increase at a CAGR of 4.2% from 2026 to 2033 (https://www.marketresearchintellect.com/product/global-synthetic-dyes-and-pigments-market/).

Most naturally occurring colors are produced by bacteria, fungi, and algae, which synthesize a wide range of pigments, including quinones, carotenoids, melanins, and many more (Table 1) [6]. Globally, the pharmaceutical, cosmetic, food, textile, painting, and plastic industries are seeing a sharp rise in demand for naturally derived pigments [7]. Although plant-based natural dyes are currently available, some drawbacks must be considered, including the high cost, batch unrepeatability, easy denaturation by pH changes, low productivity, and restriction to specific seasons to cultivate target plants [8]. Thus, microbes are being studied extensively as a potential tool for pigment synthesis in order to address the global demand for natural pigments [9]. Furthermore, significant advancements have been made lately in the synthesis of microorganism-based pigments, and certain pigments have been generated in big companies, such as DSM Company, which produces β-carotene, and Vitatene Company, which produces lycopene [8].

Bacterial fermentation stands up as particularly favorable, considering its natural and safer profile compared to typical chemical synthesis processes [10]. There are now just a few bacterial pigments that have been approved by European Food Safety Authority and the US Food and Drug Administration, such as indigo, riboflavin, β-carotene, lycopene, and astaxanthin [11]. Notably, some carotenoid is only permitted for use in animal consumption and not for human use. For instance, *Paracoccus carotinifaciens*’ astaxanthin is already on the market as a natural substitute to satisfy the rising demand for astaxanthin. But it’s crucial to remember that this bacterium is only permitted for use in animal feed [12].

This current review provides a comprehensive and up-to-date data of the main carotenoids from bacterial sources, presenting detailed information about laboratory screening of carotenoid-producing bacteria, production enhancement via optimization of physicochemical parameters or via genetic modification strategies, fermentation strategies and the extraction procedures. Additionally, the current review discussed the recent biological application of bacterial-derived carotenoids, showing that bacteria are the most valuable sources for industrial production of natural carotenoids.

**Table 1:** The main classes of microbial pigments

Class	Examples	References
Carotenoids	Carotenes–xanthophylls	[6]
Melanines	Eumelanin–pheomelanin–allomelanin	
Riboflavins	Riboflavin	
Phycobiliproteins	Phycocyanin–phycoerythrin–allophycocyanin	
Pyridones	Indigoidine	
Prodigioninies	Prodigiosin–heptylprodiogiosin–undecylprodigiosin	
Quinones	Anthroquinones–naphthoquinones	
Oxyindoles	Violacein–deoxyviolacein–oxyviolacein	

## Bacteria are superior cell factories for the production of carotenoids

Carotenoids are naturally found in plants and microorganisms, but since animals and humans cannot synthesize them by de-novo, they must receive them through diet [13]. The bulk of naturally occurring carotenoids originates from plants, as they are extensively distributed in flowers, leaves, vegetables, seeds, fruits, and roots [14], and β-carotene is the most commonly derived carotenoid from plants [15]. Apart from plants, diverse microbiological organisms, such as algae, bacteria, and fungi, can also store different types of carotenoids within their cells as metabolic byproducts [16]. When compared to plant sources, microbial fermentation offers many benefits for product manufacturing, including a better yield, quicker extraction, cheaper production, no seasonal variation, availability of raw materials, and a high degree of degradability [17].

Bacteria are the most easily manipulated microorganisms with the ability to produce a variety of carotenoids with high growth rates, minimal nutritional requirements, and cheaper production costs [18]. The main sources of natural astaxanthin are microalgae and red algae, but their supply is restricted and varies seasonally [19], and water pollution can also have a detrimental effect on the production of astaxanthin in algae [20]. Moreover, due to their rapid growth and ease of downstream extraction, bacteria are thought to be superior models for the industrial production of astaxanthin [21]. In addition, extracting zeaxanthin from algae is a complex and costly process, with challenges including waste generation, low extraction efficiency and yield, and instability; therefore, straightforward bacterial production of zeaxanthin has gained interest [22].

When it comes to producing pigments, bacteria are superior to fungi in a number of ways, such as having a shorter life cycle, being easier to genetically modify, and lower production cost [23]. In addition, the co-production of mycotoxins restricts the industrial use of fungi [24]. Furthermore, the short incubation time of bacteria, generally 4 days, to obtain carotenoids may be favorable to maintain their stabilities [25], as the main obstacle in carotenoids production is their sensitivity to light, water activity, heat, acidity, and air [17]. Vatanyoopaisarn et al. (2024) state that because of their quick cultivation times, bacteria are microorganisms of great interest for the production of ꞵ-cryptoxanthin [4]. On the other hand, the production of carotenoids by algae, such as *C. minutissima*, occurred after 30-day cultivation [26].

Additionally, bacterial cells are much easier to break than yeast or microalgae cells, which have more complicated and stronger cell walls. This facilitates the extraction of carotenoids from bacteria and lowers the extraction cost [27]. Furthermore, compared to eukaryotic algae and plant systems, the prokaryotic origin of bacteria facilitates the production of genetic modifications to enhance the production of carotenoids [28]. On the top of that, in the Carotenoid Database (http://carotenoiddb.jp/index.html), over 1200 carotenoids and carotenoid precursors have been identified so far from 722 organisms. Of these, 324 are sourced from bacteria, and 251 carotenoids are unique to these microbes [29]. These traits suggest that the field of bacterial pigments holds promise for innovative biotechnological uses and a promising avenue for the commercial production of natural carotenoids (Table 2) [23].

**Table 2:** Comparison between natural sources of carotenoids

Source	Examples	Characteristics	References
Plant	Pumpkin Tomatoes Carrots	Advantages: Appeal to the consumer, and a wide range of tones Disadvantages: It is hard to characterize and standardize because of differences in agricultural practices and climatic conditions The stability and effectiveness of these pigments are restricted, particularly when subjected to extreme pH, temperatures, and light alterations, high cost, and wide cultivation area	[14, 17, 27]
Bacteria	*Rhodococcus maris* *Micrococcus roseus* *Gordonia jacobaea*	Advantages: Bacteria produce nearly all forms of carotenoids, including C-50, C-45, C-40, and C-30 carotenoids, a source of rare carotenoids, short-cycle, reduced media requirements, high production efficiency, do not compete for arable land, and are easy to control contamination Unaffected by climate, easy to genetically engineer due to prokaryotic nature, assisting in pollution prevention by utilizing agro-industrial waste as a substrate, potentially useful source for industrial usage, high stability due to short cultivation period, and low cost compared to plants, algae, and fungi Disadvantages: The manufacturing expense is still far greater than that of artificial sources	[14, 17, 18, 21, 22, 25, 27]
Algae	*Haematococcus* sp. *Chlorella* sp. *Dunaliella salina * *Spirulina*	Advantages: Due to their high capacity to accumulate carotenoids under certain stress circumstances, they make an excellent cell factory for carotenoid synthesis Easy to cultivate Disadvantages: Climate limitation, naturally slow development, high production cost, low yield, risk of bacterial and protozoal contamination, and limited production capacity	[14, 19, 20, 22, 27]
Fungi	*Phaffia rhodozyma* *Blakeslea trispora* *Mucor circinelloides*	Advantages: A wide variety of nutrients, including potato extracts and maize stalk hydrolysate, may be used, short-cycle, unaffected by climate, and with high production efficiency Disadvantages: Co-production of mycotoxins restricts their industrial use and high production costs	[14, 23, 24, 27]

## General characteristics of natural carotenoids

Carotenoids are chemically defined as lipophilic substances that are insoluble in water, having a chromophore with a maximum absorbance between 400 and 500 nm, which accounts for their distinctive yellow to reddish hues [30]. Carotenoids exhibit considerable variation in their absorption maxima and fine structure, as well as, the ratio of absorption peak heights between peaks II and III is utilized to differentiate carotenoids and their isomers. For example, the absorbance peaks of lycopene, a red acyclic molecule, are at 444, 470, and 502 nm due to its 11 conjugated double bonds. β-Carotene, on the other hand, exhibits an orange color with a maximum absorbance at 422, 445, and 473 nm due to its 10 conjugated double bonds in its cyclic portion [30, 31].

**Fig. 1:**
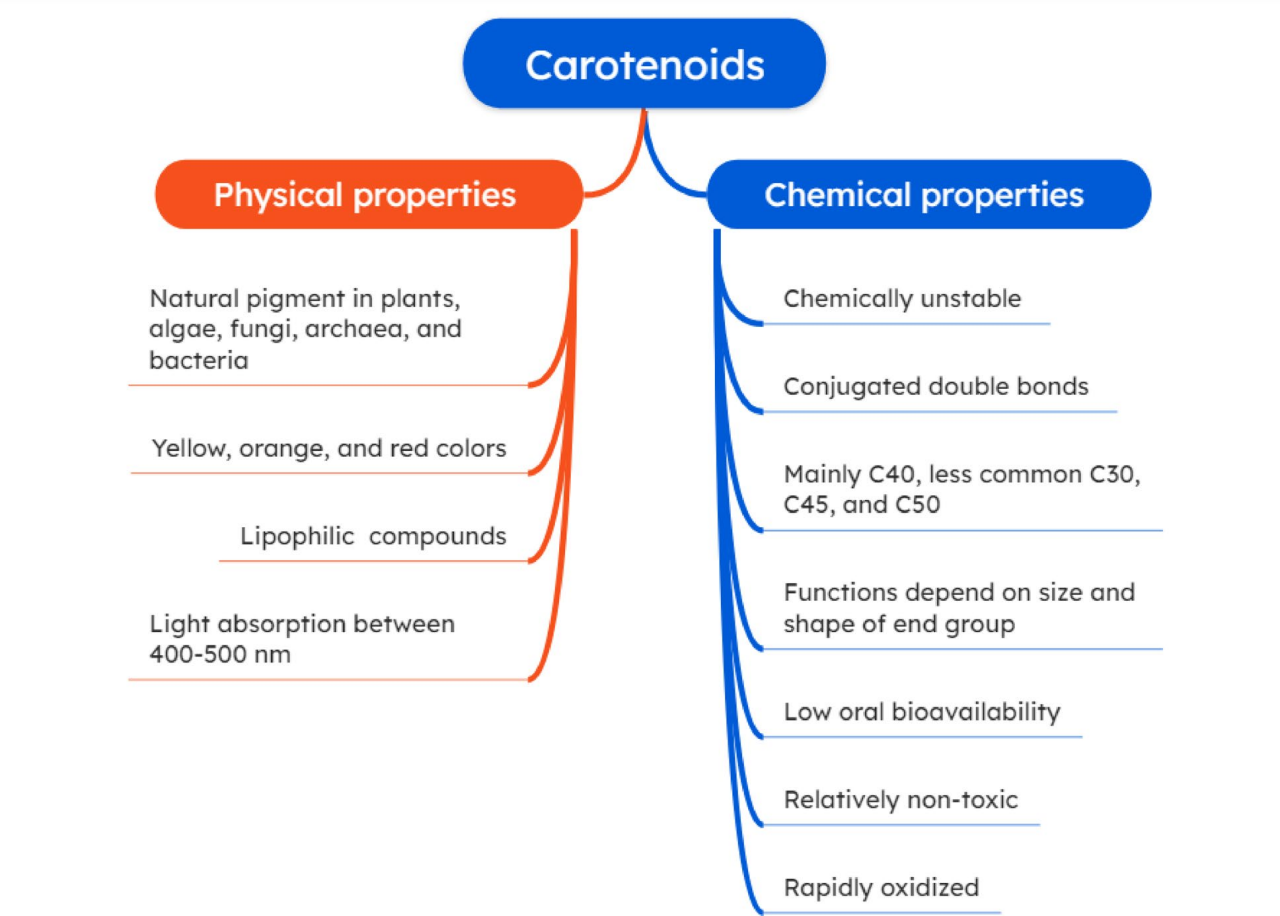
Physicochemical properties of bacterial carotenoids

The conjugated double bond system is the most significant structural feature of these compounds and the main cause of their physicochemical characteristics (Fig. 1) [32]; at least seven conjugated double bonds are essential for gaining a colored carotenoid [33]. In 1906, the first carotenoids were separated by chromatography, and in 1930, the structures of lycopene and β-carotene were determined. There are currently 1204 carotenoids known to exist in natural sources, and the majority of these are made up of 40-carbon chains (1121), which are followed by C50 (37), C30 (33), and C45 (13) [34].

These pigments are divided into two primary groups: carotenes (hydrocarbon carotenoids) and oxygenated carotenoids (also known as xanthophylls) [35]. Carotenes are non-polar hydrocarbons with a simple structure, which do not contain oxygenated functional groups. On the other hand, the oxygen atom in xanthophylls can take various forms, such as hydroxyl (–OH), carbonyl (C=O), a combination of hydroxyl and carbonyl groups, or esters of hydroxyl groups [35].

## Biosynthesis of bacterial carotenoids

**Fig. 2:**
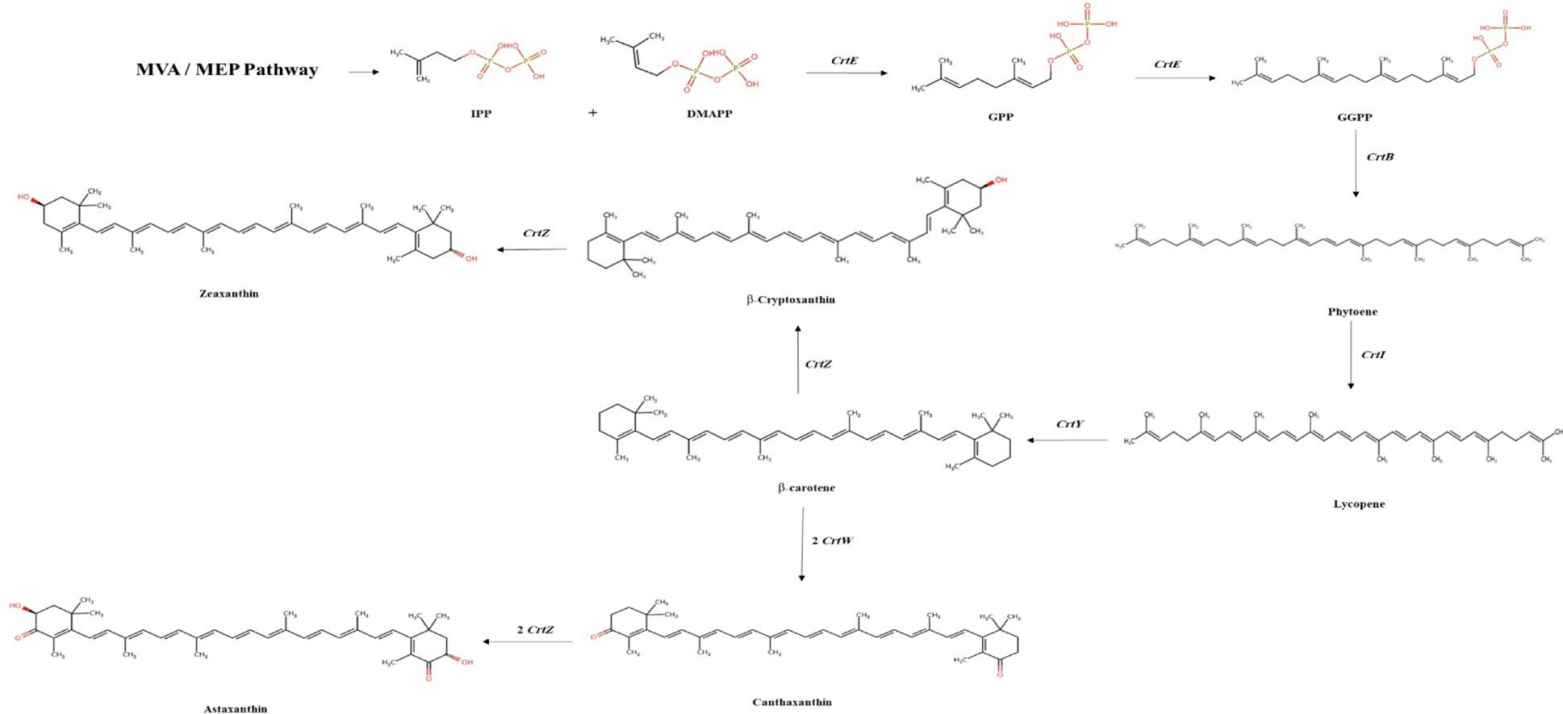
Scheme of the biosynthesis of bacterial carotenoids using the MVA/MEP pathway. *MVA* mevalonate pathway, *MEP* methylerythritol-4-phosphate pathway, *IPP* isopentenyl pyrophosphate, *DMAPP* dimethylallyl pyrophosphate, *CrtE* prenyl transferase, *GPP* C10-geranyl diphosphate, *GGPP* C20-geranylgeranyl diphosphate, *CrtB* phytoene synthase, *CrtI* phytoene desaturase, *CrtY* lycopene cyclase, *CrtZ* β-ring hydroxylase, and *CrtW* β-carotene ketolase

Most bacterial carotenoids have a C40 skeleton, such as β-carotene, β-cryptoxanthin, zeaxanthin, canthaxanthin, and astaxanthin [36]. Their biosynthesis starts from the C5 isoprenoid known as isopentenyl pyrophosphate (IPP) and its isomer dimethylallyl pyrophosphate (DMAPP), and these precursors are formed through the mevalonate pathway (MAP) or the methylerythritol-4-phosphate pathway (MEP) [36]. Initially, a head-to-tail condensation reaction occurs between one IPP molecule and one DMAPP molecule, producing C10-geranyl diphosphate (GPP) [37]. Secondly, chain elongation occurs during successive cyclical reactions and is catalyzed by prenyl transferase (CrtE). Two IPP molecules are combined with GPP, which produces C20-geranylgeranyl diphosphate (GGPP), and two units of the produced GGPP are combined to produce the C40 chain [37]. The first colorless C40 carotenoid, phytoene, is the product of two molecules of GGPP formed by tail-to-tail condensation, losing two diphosphate groups, which are catalyzed by phytoene synthase (CrtB) [38]. Subsequently, elimination, addition, substitution, and rearrangement reactions produce diverse carotenoids [38]. Lycopene is a colored carotene created from four consecutive desaturation reactions of the phytoene molecule by the action of phytoene desaturase (CrtI). Through cyclic reactions by lycopene cyclase (CrtY), lycopene produces α-carotene and β-carotene. A hydroxylation reaction in one of the β-carotene rings produces a β-cryptoxanthin molecule by β-carotene hydroxylase (CrtZ), where zeaxanthin is formed by two hydroxylation reactions [37]. Canthaxanthin is biosynthesized from the precursor, β-carotene, through the action of a β-carotene ketolase enzyme (CrtW), and astaxanthin is synthesized by the successive action of two enzymes: CrtZ and CrtW (Fig. 2) [37]. After their biosynthesis, carotenoids are localized into the phospholipid bilayer in different ways, depending on their polarity and functional groups, to perform their physiological function as mentioned above. Deep within the hydrophobic core of the biomembrane reside apolar carotenes, such as lycopene and β-carotene. However, polar xanthophylls, such as astaxanthin, seem to extend their polar functional groups towards head group areas, allowing them to bridge into the phospholipid bilayer [39].

## Bioproduction of the fundamental bacterial carotenoid

**Fig. 3:**
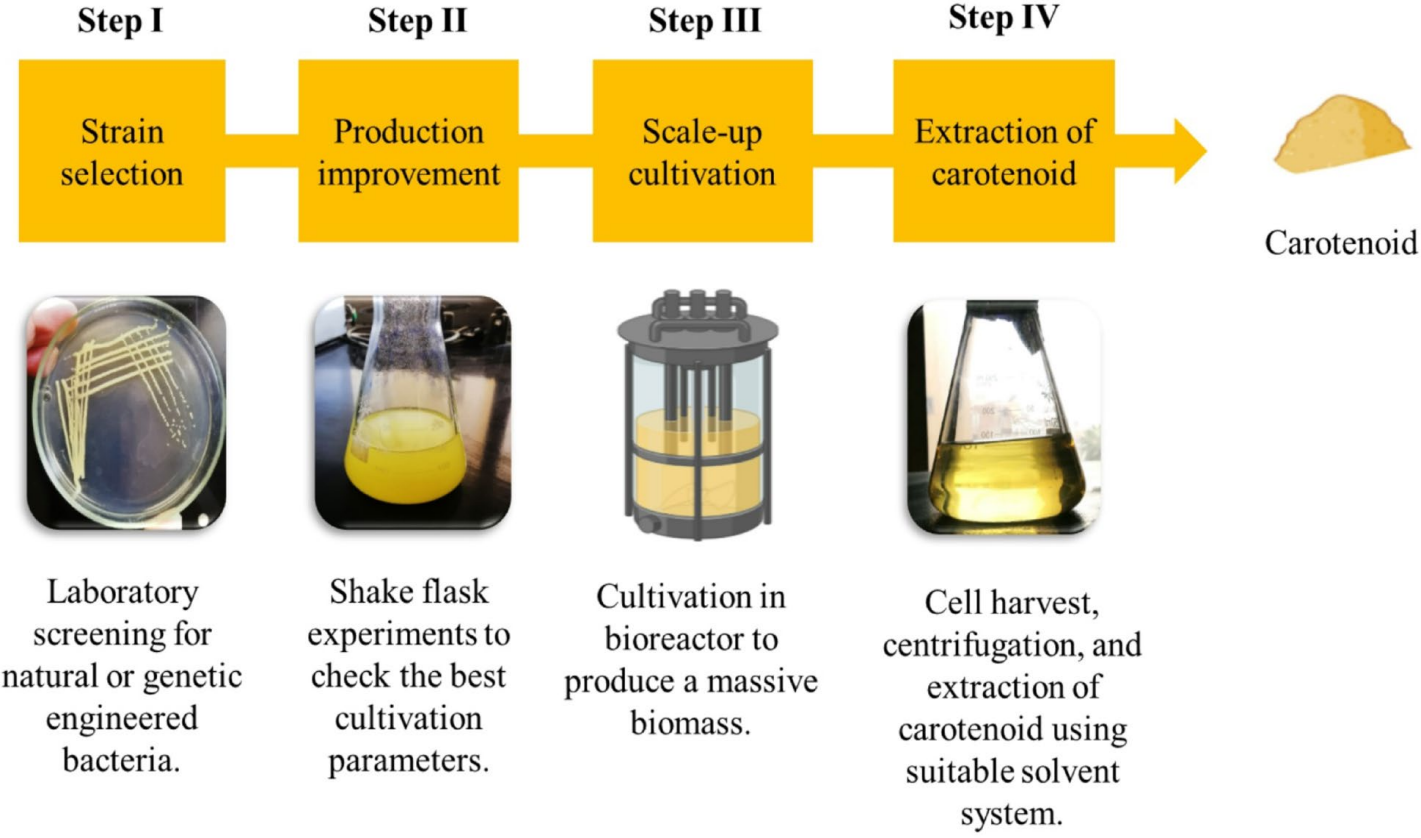
Bioproduction steps of bacterial carotenoid

Bioproduction is the process of creating valuable biological products or chemicals utilizing living cells, organisms, or biological systems, providing a sustainable substitute for conventional chemical manufacturing [40]. The bioproduction of carotenoids passes through four main steps, as illustrated in Fig. 3.

## Step I: laboratory screening of carotenoid-producing bacteria

**Fig. 4:**
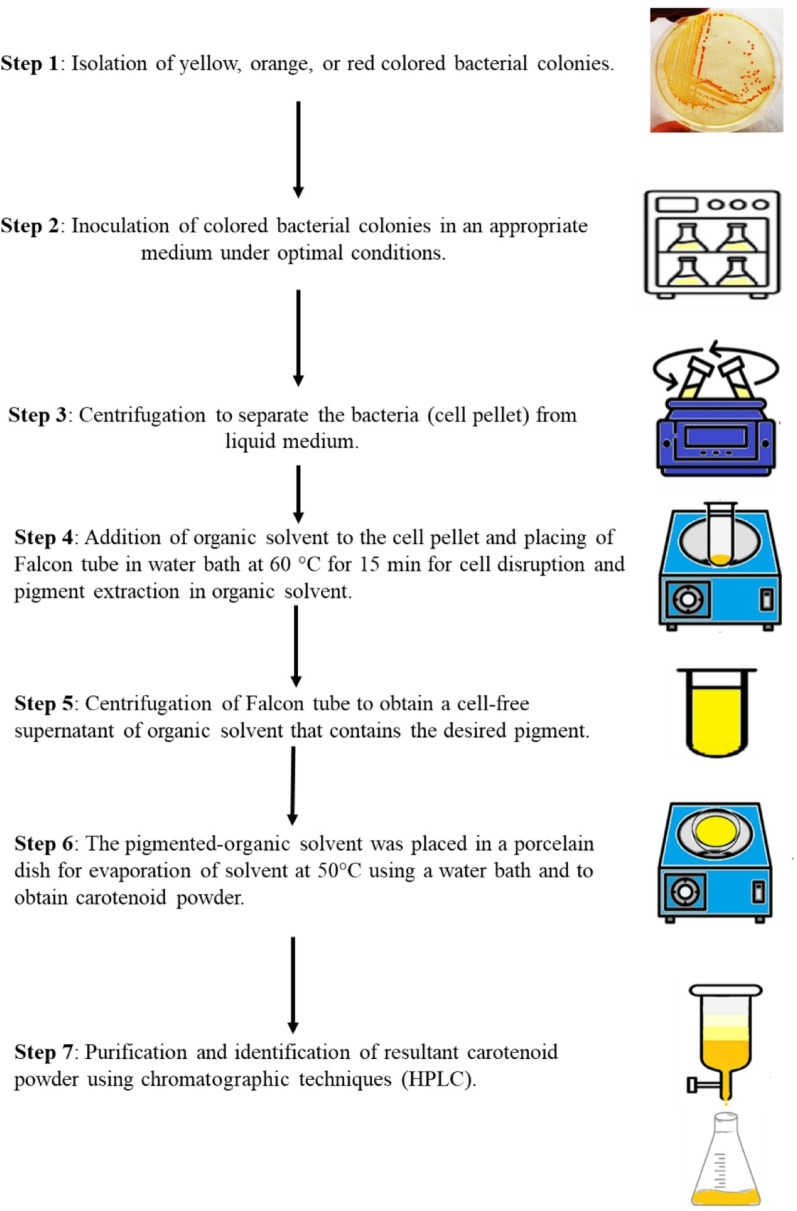
An illustrative laboratory workflow for screening the carotenoid-producing bacteria (not a universal method)

The preliminary screening of carotenoid-pigment-producing bacteria occurs through two steps. The first step is the visual investigation of yellow-orange or red cell-bound pigmented colonies that are grown on an appropriate agar medium, followed by the isolation and purification of these colored colonies using the standard microbial manipulation technique. The second step involves the extraction, purification, and characterization [41]. For example, the screening of carotenoid pigment production by *Micrococcus luteus* and *Staphylococcus aureus* using the preliminary screening approach (Fig. 4) [25, 42]. Nonetheless, there is no universal laboratory workflow for screening the carotenoid producting bcateria.

Numerous advancements have been made in the quick and easy identification of microbial pigments. A portable Raman spectrometer that uses an excitation laser at 532 nm to detect pigments is one of the better examples [23]. This cutting-edge method allows for the rapid identification of compounds even in extremely complex mixtures, with the aid of spectroscopic techniques (nuclear magnetic resonance, high-performance liquid chromatography, mass spectrometry, liquid chromatography-mass spectrometry, and ultraviolet–visible spectroscopy), without the need for individual compound purification [23]. By using 1 H and 13 C nuclear magnetic resonance and circular dichroism spectroscopy, it was possible to identify the carotenoid production by *Algoriphagus confluentis* NBRC 111,222 T and *Algoriphagus taiwanensis* JCM 19755, which showed that the pigments were blends of monocyclic carotenoids [43].

Another method for screening carotenoid synthesis is genome sequencing [4]. To validate the carotenoid synthesis pathway, the whole genome sequencing of *Pantoea anthophila* FL1_IS5 was examined, and the existence of carotenoid biosynthesis genes (*crtE*, *crtB*, *crtI*, *crtY*, *crtZ*, and *crtW*) was discovered [4]. The full astaxanthin pathway found in *Sphingomicrobium aquimarinum* sp. nov. was also using comparative genomic analysis [21]. In addition, the genome mining of a novel zeaxanthin-producing bacterium, *Sphingomonas* sp. strain BN140010 T, allowed for the identification of the zeaxanthin biosynthesis gene cluster and zeaxanthin production [44]. Furthermore, the presence of the *crtY* gene in *Paracoccus haeundaensis* SAB E11 was used to determine the carotenoid production [45]. The most common bacteria that produce carotenoids are listed in Table 3.

**Table 3:** Production sources of bacterial carotenoids

Source	Characteristics	Carotenoid	Bacterial source	References
Natural source	Advantages: More natural, safer, widely accepted by consumers, and low cost of production. Disadvantages: Insufficient production and cannot satisfy the demands of the industry.	β-Carotene	*Sphingobacterium antarcticus-Sphingomonas* sp.*-Flavobacterium multivorum-Acropora nasuta-Holothuria scabra-M. roseus-Exiguobacterium acetylicum* S01*-Nodosilinea antarctica* LEGE13457*-Cyanobium gracile* LEGE12431*-Bacillus subtilis* PD5*-Brevibacterium* sp. strain KY4313*-Lichenibacterium ramalinae-Lichenibacterium minor-Corynebacterium glutamicum-Jeongeuplla avenae-Citricoccus parietis* strain AUC*s-Pseudomonas putida*	[46–60]
		Astaxanthin	*Brevundimonas* sp. SD212*-P. carotinifaciens* E-396*-Brevundimonas* spp. (wild type)-*Paracoccus bogoriensis-Mycobacterium lacticola-Brevundimonas scallop-E. acetylicum* S01*-Gordonia alkanivorans-P. carotinifaciens-Microbacterium* sp. LEMMJ01	[54, 60–68]
		Zeaxanthin	*Synechocystis* sp. PCC 6803*-Sphingobacterium antarcticus-Sphingobacterium multivorum-Chryseobacterium proteolyticum* 9670 (NR_112113)*-Mesoflavibacter zeaxanthinifaciens-Synechococcus elongatus* PCC 7942*-Gramella planctonica-Alkalinema pantanalense* LEGE15481*-C. gracile* LEGE12431*-Leptolyngbya* sp. LEGE13412	[28, 46, 48, 53, 54, 69– 72]
		Canthaxanthin	*G. jacobaea* MV-1*-Dietzia natronolimnaea* HS-1*-M. roseus-Dietzia schimae* YIM 65,001*-Cuspidothrix issatschenkoi* LEGE03282*-Chryseobacterium* sp.*-G. alkanivorans-Brevibacterium* sp. *strain* KY4313*-Bradyrhizobium* spp.*-Lactobacillus pluvialis-R. maris*	[51, 53, 56, 60, 67, 68, 70, 73– 75]
		β-Cryptoxanthin	*Erythrobacter longus* strain OCh 101*-Brevibacterium linens-S. antarcticus-H. scabra-Celullophaga fucicola* 416*-P. anthophila* FL1_IS5 strain	[46, 50, 75– 78]
Genetic engineering	Advantages: Fast production, short fermentation cycle, production of carotenoid of interest (single product), and not restricted by geography or environment. Disadvantages: Complex development and high cost of production.	β-carotene	*Escherichia coli* strain Nissle 1917-*E. coli* BL21 (DE3)-*Zymomonas mobilis*	[79–81]
		Astaxanthin	*Synechocystis* sp. PCC 6803-*Brevundimonas* sp. M7-*Agrobacterium aurantiacum*-*C. glutamicum*	[29, 82–84]
		Lycopene	*Lactococcus lactis-E. coli-Rhodobacter sphaeroides*	[57, 85, 86]
		Zeaxanthin	*S. multivorum*	[28]

## Step II: enhancement productivity of carotenoids from bacterial sources

### A. Optimization of physicochemical parameters using one-factor-at-a-time (OFAT) approach

The carotenoid pigments are produced under the control of various factors, including pH, type of medium, incubation period, temperature, and other factors [16]; therefore, in order to get strategies that motivate microorganisms to adjust carotenoid synthesis, it is crucial to understand cultivation parameters [87]. In the OFAT method, one variable is altered while all other variables remain unchanged in order to see how it affects the result. This method, sometimes referred to as monothetic analysis, enables the sequential testing of distinct elements to pinpoint important drivers of the production process. A major disadvantage of OFAT is that, despite its ease of use and ability to quickly offer information about a single element, it is unable to identify interactions across factors, which may result in incomplete optimization and sometimes misleading findings [88].

#### Growth medium and inoculum size

The best fermentation medium depends on the strain. According to Nosair et al. [42], the brain heart infusion medium was accompanied by the highest carotenoid production by *S. aureus* A2 isolate, which was 1.7 times higher than that of the glucose broth medium and 1.6 times higher than that of the peptone water medium. According to Hagaggi and Abdul-Raouf [59], the optimum growth and pigment production by *C. parietis* AUCs were achieved in nutrient broth. From the result of the OFAT experiments, it was observed that the tryptic soya broth was associated with the highest productivity of carotenoid pigment by *M. luteus* [25]. According to Rezaeeyan et al. [89], the tryptic soya broth medium was the preferred medium for carotenoid production by the *Kocuria* sp. strain QWT-12. In addition, the production of carotenoid pigment by *B. subtilis* was done using tryptic soya broth, as described by Filluelo et al. [90]. This may be due to the components of the medium that needed for the pigment’s production [91].

The inoculum size greatly affected the carotenoid production; the production of astaxanthin and zeaxanthin by *Paracoccus marcusii* RSPO1 was significantly affected by inoculum size [92]. The optimal inoculum size for the production of carotenoids by *Halorubrum ruber* was 8.7% [93]. A significant 2.76-fold increase in astaxanthin and a 10.14-fold increase in zeaxanthin were achieved during an inoculum size of 10% [94]. In another study, Fatima et al. [95] reported that *Micrococcus* species produced more pigment with an inoculum size of 2%.

#### Carbon and nitrogen source

The source of carbon is crucial for both biomass development and carotenoid accumulation, but the preferences of specific microbial strains will also influence the efficacy of a given substrate [96]. The higher productivity of astaxanthin was observed when glucose, sucrose, and fructose were utilized as a carbon source [97]. In another experiment, the astaxanthin output was high when raffinose and galactose were used [98]. Methane was also utilized for the production of canthaxanthin by *Methylococcus capsulatus* [99]. The highest production level of total carotenoid was 1.33-fold when glucose was replaced with sucrose [93]. According to Nosair et al. [42], mannitol was the most effective carbon source.

Yeast extract and peptone are preferred by bacteria as an organic nitrogen source. Numerous studies demonstrate that these substrates produce the highest biomass and a high carotenoid yield [98]. Replacing yeast extract with other nitrogen sources resulted in significantly decreased production levels of total carotenoid [93]. In the study conducted by Al-Monofy et al. [25], the best nitrogen source for carotenoid pigment production was tryptone. However, cheap inorganic salts like potassium nitrate, urea, ammonium chloride, and ammonium sulfate are also effectively utilized to lower the cost of the fermentation medium [100]. *Arthrospira platensis* produced more carotenoids when sodium nitrate was added to the medium at a concentration of 0.1 gm/L, while the greatest carotenoid yield was obtained when urea was added [11].

According to Hwang et al. [93], the ideal carbon/nitrogen (C/N) ratio for carotenoid formation might range between species and even within the same species, depending on growth phases and special environmental factors. The largest production of β-cryptoxanthin at 10.59 mg/L was obtained with 10 g/L of glucose and 5 g/L yeast extract in nutritional broth medium (C/*N* = 2/1) [4]. *Haloferax mediterranei*’s carotenoid synthesis revealed the greatest results with a low C/N ratio (0.5% glucose and 100 mM nitrate, approximately a 1.4:1 of C/N ratio) [93].

#### Temperature and pH

It is well established that pH and temperature are critical parameters for the effective growth of microorganisms, and that their optimal levels can enhance the synthesis of specific cellular metabolites [101]. The optimal pH and temperature for carotenoid production differ based on the variety of microorganism and carotenoid. It is possible that the ideal pH and temperature will vary even within the same species when employing different substrates that necessitate distinct enzymatic conversions, as temperature and pH are crucial factors for enzymatic reactions [102]. Several sources indicate that carotenoid-producing bacteria are grown at a pH of 5.5–7.5 and a temperature of 25–37 °C [103]. Al-Monofy et al. [25] reported that the paramount temperature for carotenoid production was 37 °C. In addition, Fatima et al. [95] discussed that the best temperature for the production of *Micrococcus* species’ pigment was 37 °C. While, the optimum temperature for carotenoid production by *Kocuria* sp. RAM1 was 31.7 °C [104], and a temperature of 30 °C was the best for carotenoid production by *Paracoccus zeaxanthinifaciens* ATCC21588 [105]. On the other side, the essential pH for augmenting the production of carotenoid pigment was 7 [25]. Moreover, according to Korkerd et al. [106], the superlative pH for carotenoid production by *P. anthophila* FL1_IS5 was 7.4.

#### Light and UV radiation

Numerous studies have investigated how light and UV radiation exposure enhance carotenoid production as a natural defense mechanism against photo-oxidative stress [107]. In addition to determining their colors, the conjugated double-bond chain of carotenoids functions as a light-absorbing chromophores that have significant biological significance in shielding cells from the damaging ageing effects of UV light and providing antioxidant properties [108]. In fact, the cells of the bacterium *D. natronolimnaea* exhibited increased biomass growth and carotenoid synthesis when exposed to white light [109]. In a glucose and sodium sulfate-containing environment, it has been demonstrated that white light significantly influenced the production of astaxanthin, lutein, and canthaxanthin in the biomass of *G. alkanivorans*, but that switching out these substrates diminished the effect of light [110]. According to Mohanty et al. [111], *Methylobacter* sp N39’s production of β-carotene was triggered by a 10-min exposure to UV light.

##### Glycerol and fatty acids

The production of carotenoids is impacted by glycerol and oils [103]. Especially, the use of crude glycerol produced a greater quantity of carotenoids than that of pure glycerol, due to the raw glycerin’s fat content following biodiesel production [100]. The addition of glycerol (0.5%) significantly enhanced the production of β-carotene by *M. luteus* [25]. According to Kot et al. [112], the carotenoid production in recombinant *E. coli* was increased (~ 3.3-fold) after adding glycerol. In addition, it was found that the addition of glycerol resulted in an elevation in carotenoid concentration by 7.5-fold in *Rhodococcus opacus* PD630 [113]. Further, the addition of fatty acids to a medium containing just glycerol confirmed this hypothesis, since the researchers determined that linoleic, stearic, palmitic, and oleic acids doubled carotenoid output [100].

#### Agitation, aeration, and incubation time

The incubation of isolates under shaking conditions increased carotenoid pigment production [25]. Mantzouridou et al. [114] examined how agitation affected carotenoid production and discovered that it increased β-carotene production because the oxygen requirement of a fermentation procedure was satisfied by agitating the broth. Also, the agitation kept the culture’s physical and chemical conditions uniform through constant mixing [114]. In addition, the fermentation medium must be sufficiently aerated [115]. The need for oxygen during fermentation can be met by adjusting the medium’s working volume in the flasks and mixing the flasks on an orbital shaker or by combining the oxygen supply with the medium in a bioreactor [116]. The influence of dissolved oxygen was studied on zeaxanthin by *Flavobacterium* genus, and it was found that 10% pO_2_ was the best condition for zeaxanthin production in a batch bioprocess, reaching a total carotenoid concentration of 3280 µg/L, with 86% of zeaxanthin [117]. Moreover, the carotenoid pigment yield was measured over 5 days at 24-h intervals to evaluate the impact of incubation time, and the data showed that on day 4, the yield of carotenoid pigment was at its greatest [25]. Also, Kandasamy et al. [91] discovered that *M. luteus* produced pigment progressively from day 1 to day 4, and then after 4 days of incubation, there was a small decrease in pigment production.

#### Tricarboxylic acid (TCA) cycle intermediates and trace elements

Numerous studies have shown that the addition of TCA intermediates like α-ketoglutarate, pyruvate, citrate, d-isocitrate, malate, fumarate, and succinate to the fermentation medium has a beneficial effect on carotenoid production [103]. TCA contributes to the formation of the carbon skeleton of carotenoids and serves as a source of energy for their synthesis [118]. Zeaxanthin production in *F. multivorum* biomass was reported to increase 6-fold when α-ketoglutarate, malic acid, and isocitric acid were introduced to the medium [48]. The anaerobic carotenogenic bacterium *Cellulosimicrobium* AZ increased carotenoid yield to 28.86 mg/L due to the presence of succinate, citrate, glutamate, and malate in the medium [119]. Furthermore, fumaric acid and malic acid were found to be strong enhancers of carotenoid biosynthesis in *P. marcusii* RSPO1 [92].

In several bacteria, magnesium, zinc, copper, and iron have been mentioned as carotenoid inducers [103]. In a fed-batch fermentation of *D. natronolimnaea*, the addition of Zn^2+^ (27 ppm), Fe^3+^ (30 ppm), and Cu^2+^ (28.75 ppm) considerably boosted the production of total carotenoids (9.7 mg/L) [120]. In a different study, the maximum total carotenoid yield of 413 mg/L for *Rhodopseudomonas faecalis* PA2 was obtained by adding 0.05% of Fe^3+^ to the medium, while the yield under unoptimized conditions was 190 mg/L [121].

#### Oxidative stress and osmotic stress

The production of carotenoids is also impacted by oxidative stress. The proteins, DNA, and cell membranes of bacteria are harmed by oxidative stress. To prevent this, microbial pigments use their free radical scavenging capabilities to neutralize free radicals and donate electrons to them, delaying or preventing cellular damage [122]. The medium’s addition of 10–20 mM H_2_O_2_ had varying effects on the accumulation of astaxanthin based on the growth stage; when H_2_O_2_ was introduced to cells in the stationary growth phase, there was a little change in astaxanthin production. In contrast, the introduction of H_2_O_2_ during the exponential growth phase only caused slight biomass inhibition but boosted astaxanthin accumulation by 60% [123]. In a separate experiment, TiO_2_ treatment (0.5 g/L) increased astaxanthin output by 2-fold in 72 h with no noticeable biomass suppression [124].

Induced osmotic stress is also a great promoter of carotenoid synthesis, according to many researchers [103]. A study examining the effects of osmotic stress produced by adding 5% NaCl to the medium on carotenoid synthesis found that the overall quantity of carotenoids increased [125]. In another investigation, adding 2% NaCl to the fermentation medium doubled the production of carotenoids [126]. The largest carotenoid production by *S. aureus* was obtained with ZnCl_2_, followed by MgSO_4_, and the lowest production was obtained with MnCl_2_ [42]. According to Hwang et al. [93], the carotenoid production was promising when the medium contained 20% and 22.5% NaCl, compared to other concentrations of NaCl.

### B. Optimization of physicochemical parameters using response surface methodology (RSM)

**Fig. 5:**
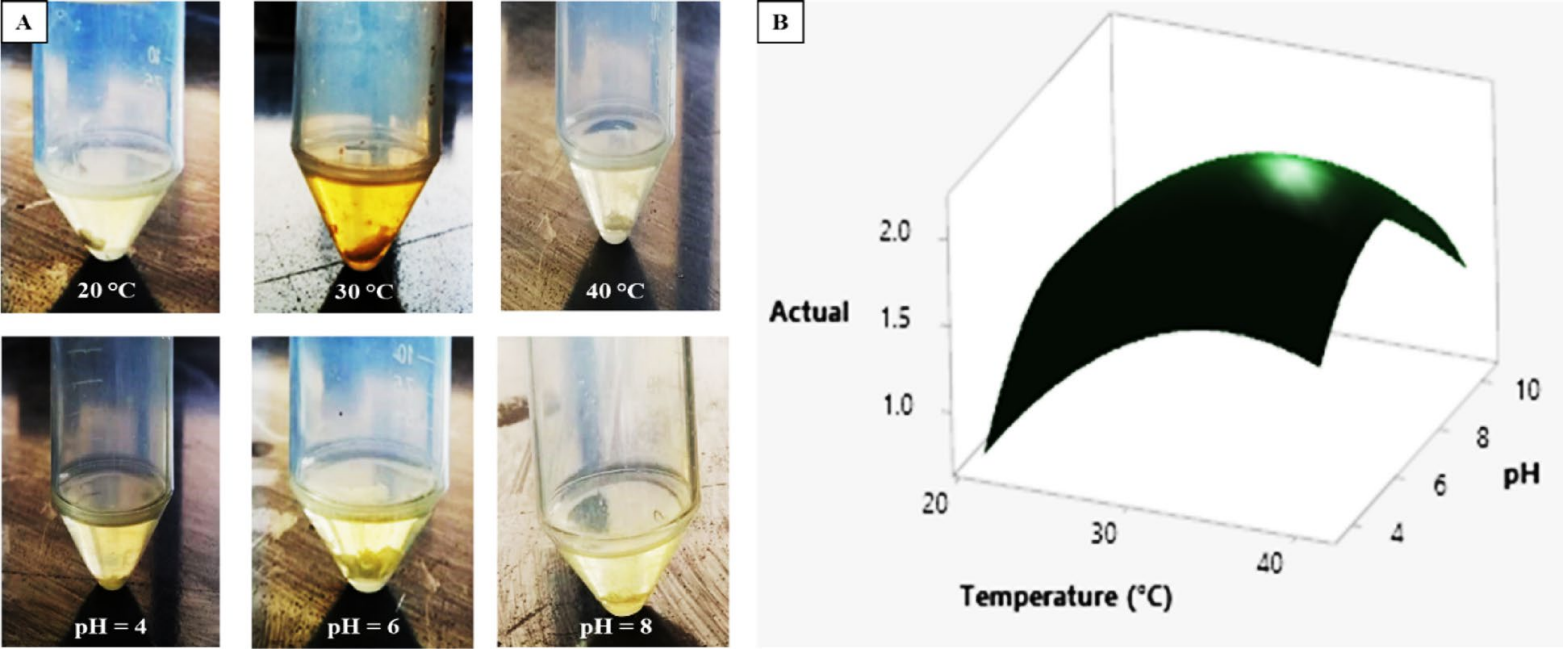
Physicochemical optimization of carotenoid pigment production using OFAT (**A**) and RSM (**B**) methodologies. In OFAT, each factor (temperature or pH) was examined at one value, and the response value (carotenoid yield) was measured. In contrast, in a time-saving RSM methodology, the effect of the interaction of two variables (Temperature vs. pH) at different values on the response value (actual) was evaluated

Using a full quadratic polynomial model to illustrate the interaction between various factors, RSM investigates the association between the response variable and other factors [127]. RSM has been extensively utilized in a number of sectors, such as agriculture, food, beverage, and medicine [127]. RSM greatly cuts down on the time and resources needed for optimization studies by allowing researchers to effectively investigate and predict the interactions between different factors and pigment yield, which eventually speeds up the creation of scalable and reasonably priced manufacturing process (Fig. 5) [128]. The production of carotenoids is frequently optimized by RSM, and the resultant optimal fermentation conditions represent the basis for the production of carotenoids in the industry after the verification step (Table 4) [127].

**Table 4:** Optimized fermentation conditions for bacterial carotenoid production using RSM

Bacteria	RSM conditions	Yield	Carotenoid	References
*Kocuria* sp. RAM1	Peptone (8.75 g/L), 31.7 °C, 180 rpm, and inoculum size (2.5%).	886 mg/L	Total carotenoid	[104]
*M. luteus*	Whey (3%), inoculum size (7.5%), 175 rpm, pH = 7, and 32.5 °C.	2190 mg/L	Total carotenoid	[129]
*P. marcusii RSPO1*	Starch (2.24 g/L), MgSO_4_ (0.416 g/L), ZnSO_4_ (0.0157 g/L), and fumaric acid (16 mM).	7.240 mg/L	Total carotenoid	[92]
*P. anthophila* FL1_IS5	Yeast extract (0.51%), carrot pomace hydrolysate (22.93%), and pH = 7.44.	31.87 mg/L	ꞵ-cryptoxanthin	[106]
*H. ruber*	37 °C, 200 rpm, yeast extract (7.96 g/L), NaCl (210 g/L), pH = 8, inoculum size (8.7%), and 9 days.	0.492 mg/L	Total carotenoid	[93]
*P. zeaxanthinifaciens* ATCC21588	Pyridoxine hydrochloride (0.18 mg/L), glucose (24.3 g/L), yeast extract (30 g/L), methyl palmitate (8 g/L), pH = 7.5, 30 °C, 180 rpm, and 3 days.	11.63 mg/L	Zeaxanthin	[105]
*S. aureus*	Mannitol (2.5%), peptone (1.5%), ZnCl_2_ (20 mM), brain heart infusion medium, pH = 5, 37 °C, and 48 h.	1.5-fold increase	Staphyloxanthin	[42]
*M. luteus*	Disodium hydrogen phosphate (5 g/L), glutaric acid (5 g/L), sodium chloride (12.5 g/L), tryptophan (12.5 g/L), tryptic soya broth medium, 120 rpm, 32.5 °C, pH = 7, 96 h, and inoculum size (2%).	1200 mg/L	ꞵ-Carotene	[25]

### Genetic engineering to improve carotenoid productivity

**Fig. 6:**
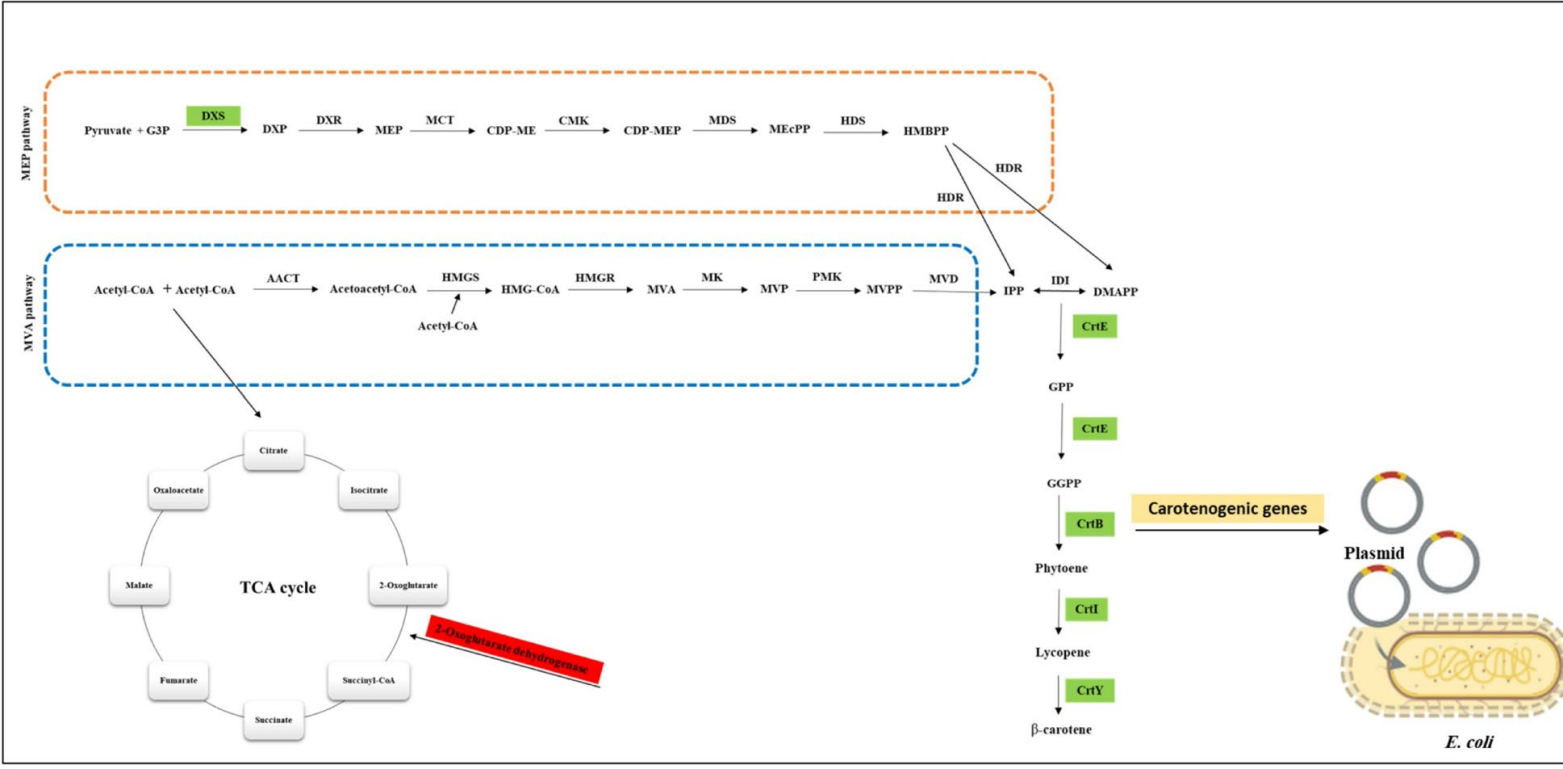
Genetic engineering to enhance carotenoid production through overexpression of carotenogenic genes that coding for key carotenogenic enzymes (green color), deletion of specific genes, such as gene that code for tricarboxylic acid (TCA) cycle 2-oxaglutarate dehydrogenase (red color), resulting in more acetyl-CoA available for MVA pathway, and heterologous expression of carotenoid synthesis genes in non-carotenogenic bacteria, such as *E. coli* (orange color). MVA: Mevalonate pathway, *MEP* methylerythritol phosphate pathway, *IPP* isopentenyl diphosphate, *DMAPP* dimethylallyl diphosphate, *HMG-CoA* 3-hydroxy-3-methylglutaryl-CoA, *MVP* 5-phosphomevalonate, *MVPP* 5-diphosphomevalonate, *AACT* acetyl-CoA acetyltransferase, *HMGS* 3-hydroxy-3-methylglutaryl-CoA synthase, *HMGR* 3-hydroxy-3-methyl-glutaryl-CoA reductase, *MK* mevalonate kinase, *PMK* phosphomevalonate kinase, *MVD* diphosphomevalonate decarboxylase, *IDI* isopentenyl diphosphate isomerase, *G3P* d-glyceraldehyde 3-phosphate, *DXP* 1-deoxy-d-xylulose 5-phosphate, *CDP-ME* 4-(cytidine 5′-diphospho)-2-C-methyl-d-erythritol, *CDP-MEP* 2-phospho-4-(cytidine 5′-diphospho)-2-C-methyl-d-erythritol, *MEcPP* 2-C-methyl-d-erythritol-2,4-cyclodiphosphate, *HMBPP* 1-hydroxy-2-methyl-2-butenyl 4-diphosphate, *DXS* 1-deoxy-d-xylulose-5-phosphate synthase, *DXR* 1-deoxy-DXR-xylulose-5-phosphate reductoisomerase, *MCT* 2-C-methyl-d-erythritol 4-phosphate cytidylyltransferase, *CMK* 4-diphosphocyt-idyl-2-C-methyl-d-erythritol kinase, *MDS* 2-C-methyl-d-erythritol 2,4-cyclodi-phosphate synthase, *HDS* 4-hydroxy-3-methylbut-2-enyl-diphosphate synthase, *HDR* 4-hydroxy-3-methylbut-2-enyl diphosphate reductase

The intricate, multi-step process of carotenogenesis involves a variety of reactions, including condensation, oxygenation, desaturation, elongation, cyclisation, hydroxylation, methylation, glycosylation, and acylation of carotenoid intermediates, which are catalyzed by the enzymes encoded by the *crt* genes (e.g., *crtB*, *crtE*, *crtI*, and *crtY*) [130]. Metabolic engineering has led to important breakthroughs to further increase carotenoid production, providing significant advantages, including quick production, shortened fermentation periods, pure final product, and freedom from environmental and spatial restrictions [10]. The ways to improve carotenoid yield via genetic engineering involve the following (Fig. 6).

#### Overexpression of key carotenoid genes in carotenogenic bacteria

One direct method to boost the production of carotenoids is to overexpress important genes that encode the enzymes involved in carotenoid biosynthesis. Both the *crtB* gene, which codes for CrtB, and the *dxs* gene, which codes for 1-deoxy-d-xylulose-5-phosphate synthase (DXS), are regarded as rate-limiting steps in carotenoid biosynthetic pathways [131]. The modified strain of *Deinococcus* bacteria produced 177.29 mg/L of deinoxanthin, 200% more than the wild-type strain, because of the overexpression of the *crtB* and *dxs* genes [131]. Furthermore, by creating plasmids that overexpressed the *crtB* and *dxs* genes, lycopene production was raised to 373.5 mg/L [132]. It is important to note that the conversion of DMAPP to GGPP by CrtE is also identified as the critical factor limiting the rate of carotenoid biosynthesis [133]. Researchers have used directed evolution methods on CrtE to increase carotenoid production in order to improve the flow of molecules along this pathway [134].

#### Deletion of specific genes in carotenogenic bacteria

By knocking down particular *crt* genes, the production of target carotenoids increased and the generation of non-target carotenoids decreased. For instance, the mechanism for converting phytoene to downstream carotenoids is cut off when the gene encoding CrtI enzyme is deleted, enabling phytoene to accumulate (phytoene concentration = 0.413 mg/L) [135]. Additionally, the modified strain produced 1.080% more phytoene (phytoene concentration = 4.46 mg/L) after the carotenoid 3′,4′-desaturase gene *crtD* was deleted and the *crtB* and *dxs* genes were overexpressed [136]. In addition, the formation of each isoprene unit requires three acetyl-CoA molecules through the MVA route, and reducing the use of acetyl-CoA in the TCA cycle through the deletion of the gene coding for 2-oxoglutarate dehydrogenase can increase acetyl-CoA flux into the MVA pathway [134].

#### Heterologous expression of carotenoid synthesis genes in non-carotenogenic bacteria

Synthetic biology research has recently focused on the use of genetic engineering techniques to exogenously express particular genes in model microbes for the manufacture of target chemicals [137]. *E. coli* has been utilized extensively as a model bacterium in a variety of biosynthetic research because of its quick growth, facile genetic modification, and obvious genetic background [137]. According to Xu et al. [138], the three genes encoding carotenoid synthetase (*crtE*, *crtB*, and *crtI*) were extracted from *Deinococcus wulumuqiensis* R12 in order to create a polycistronic plasmid. These genes were then co-expressed in *E. coli* to boost the yield of lycopene (lycopene concentration = 688 mg/L). Further, the overexpression of the *crtY* gene significantly affected the production of carotenoid, which led to the buildup of β-carotene in *E. coli* (β-carotene amount = 41 mg) [139]. In addition, the carotenoid synthesis in *E. coli* was increased by 3.5 times due to the overexpression of the *dxs* gene [140].

**Fig. 7:**
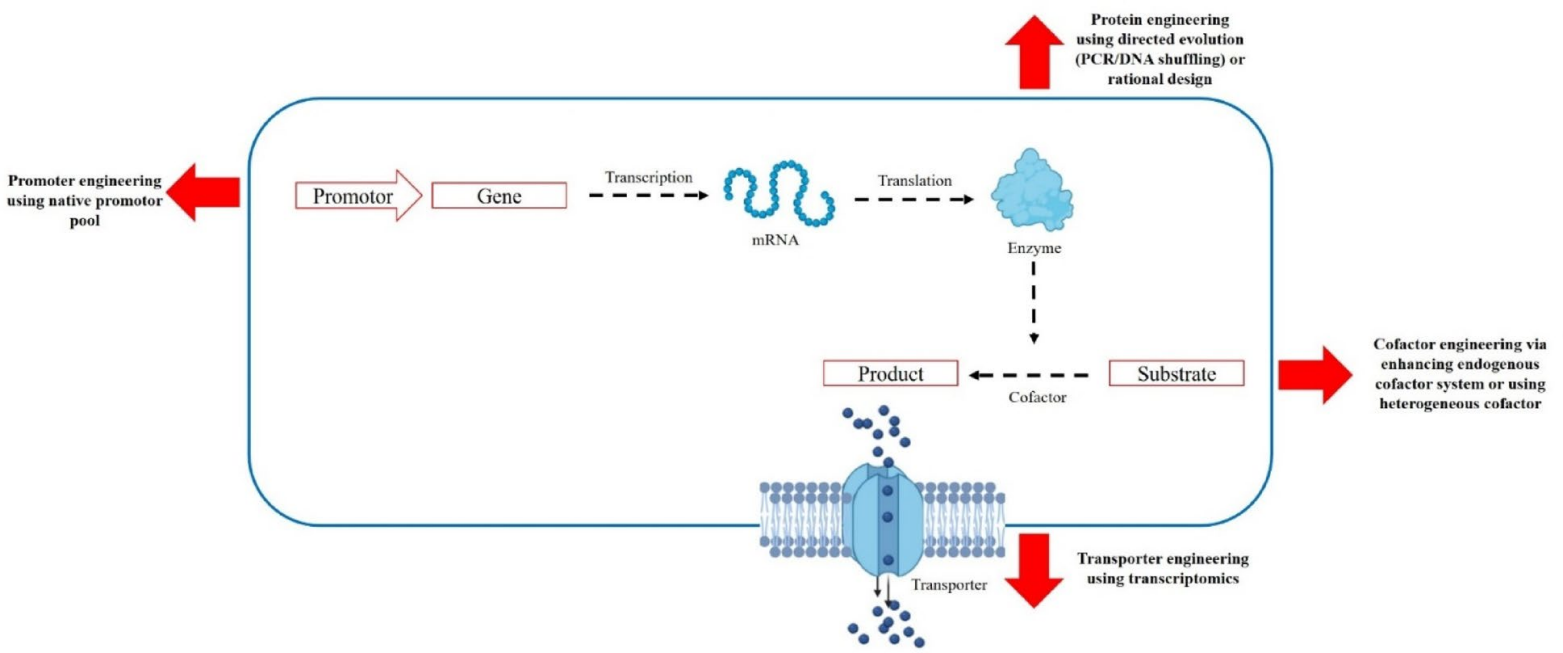
Strategies of genetic engineering to enhance carotenoid production from bacteria via promoter engineering, protein engineering, cofactor engineering, and transporter engineering

The strategies of genetic engineering to enhance carotenoid production involve the following (Fig. 7).

#### Promoter engineering

To enhance cell growth and product generation, gene expression must be precisely regulated; too much gene expression can lead to metabolic stress, while too little can result in the buildup of metabolic intermediates [141]. The most straightforward method to change gene expression by regulating the transcription level of particular genes is through promoter optimization. However, its use is contingent upon the availability of endogenous promoters with the appropriate characteristics. The promoter engineering is accomplished through screening the native promoter pools in bacteria through omics and characterized with reporters to enhance promoter sources, while synthetic promoter libraries have been created using saturation mutagenesis, site-directed mutagenesis, hybrid-promoter design, and error-prone PCR [141]. According to Durán et al. [142], substituting the original crtE promoter with the HMGS gene promoter, which encodes 3-hydroxy-3-methylglutaryl-CoA synthase, increased carotenoid synthesis by 1.43-fold.

#### Protein engineering

Enzymes are the fundamental catalytic components in the biosynthesis of natural products. Inefficient enzymes can cause feedback inhibition, undesired byproducts, and bottlenecks, which can lead to reduced productivity and synthetic efficiency [141]. Protein engineering via directed evolution accomplishes functional modifications by changing the enzyme structure, and the procedure usually involves random mutations that can be introduced into the protein through either DNA shuffling or error-prone PCR. For example, directed evolution enhanced the catalytic activity of the pathway enzymes, such as CrtE and CrtW enzymes, resulting in better astaxanthin biosynthesis [143]. Besides directed evolution, rational design is becoming more crucial in protein engineering due to the expanding availability of mechanistic and structural data [141]. In this method, engineering targets can be determined through homologous modeling, followed by molecular docking or molecular dynamics simulations, or through sequence conservatism analysis, and then followed by site-specific mutagenesis. This greatly lessens the laborious screening process that is typically needed for directed evolution. For instance, a mutation in the isopentenyl phosphate kinase enzyme, which catalyzes the transformation of dimethylallyl alcohol into the precursors of isoprenoids (IPP and DMAPP), via rational design led to an 8-fold increase in activity and 97% greater β-carotene output [144].

#### Cofactor engineering

The biosynthesis of pigments usually includes several redox reactions fueled by the NAD(P)H cofactors’ reducing power [141]. However, native cofactor systems found in microbes are frequently inadequate to support heterologous biosynthesis. A viable approach to preserving cellular redox balance is through regulating endogenous cofactor systems by increasing cofactor production or limiting cofactor usage [145]. The production of NADPH can also be increased by the addition of foreign cofactor regeneration systems, along with the regulation of endogenous cofactor systems [146]. For instance, the NADPH regeneration rate was boosted by up to 25-fold, and carotenoid production was improved by 97% by integrating a foreign Entner-Doudoroff pathway from *Z. mobilis* into *E. coli* [146].

#### Transporter engineering

A major obstacle in metabolic engineering is the extracellular secretion of naturally occurring substances, such as fat-soluble substances like carotenoids. In order to address the transport bottlenecks, many new transporters for particular pigments have been discovered in recent years and subsequently utilized [141]. The transcriptomes of two engineered strains of *Saccharomyces cerevisiae* with varying capacities for carotenoid production indicated the induction of genes related to the ABC transporter-pdr10 in the strain with high carotenoid production [147]. To verify the impact pdr10 transporter on carotenoid secretion, pdr10 from *S. cerevisiae* was introduced into *Rhodosporidium toruloides*, resulting in an increase in total carotenoid production ranging from 1.9 to 2.9 µg/ml in a bi-phasic culture [148].

## Step III: fermentation process of carotenoid-producing bacteria

Using bioreactors is one of the most crucial biotechnological processes, which provides controlled environment for growing microorganisms in order to obtain a specific bioproduct [149]. Bioreactors must be supplemented with sensors to achieve precise and real-time monitoring of the bioprocess, early problem detection, reproducibility, cost reduction, and greater efficiency [149]. Different types of bioreactors are utilized in the pigment production, such as submerged fermentation (SmF) and solid-state fermentation (SSF) bioreactors, dependent on the strain type and the prerequisite pigment (Table 5) [150]. In SmF, the microorganisms are grown in the liquid medium in a stirred tank reactor to produce the required product [150]. For instance, the production of β-carotene increased by 3.47-fold in SmF by the upregulation of carotenoid biosynthetic genes in *E. acetylicum* S01 under adjusted conditions of 1.4 g/L glucose, 26.5 g/L peptone, pH = 8.5, and temperature of 30 °C [151]. On the other hand, in SSF, bacteria are cultured on the surface of solid substrates or agro-industrial waste, which eventually reduces the cost of the medium and acts as a waste management instrument [150]. For example, the utilization of pomegranate, orange, and pineapple waste medium for the cultivation of *Rhodotorula rubra* to obtain β-carotene [152]. According to Ring et al. [120], the SSF delivers a suitable environment for the growth of microbes and simultaneously a high production yield. In addition, low water activity in SSF results in better product stability and less effluent, which raises the concentrations of the desired products and increases fermentation productivity [153].

**Table 5:** Comparison between fermentation processes for the production of carotenoids

Types	Solid-state fermentation (SSF)	Submerged fermentation (SMF)
Instrumentation		
Design	Simple bioreactor	Highly designed bioreactor
Water content	No water content	The main component of the medium is water
Inoculum size	Larger inoculum size (more than 10%)	Smaller inoculum size (less than 10%)
Production rate	Higher production rate	Lower production rate
Yield	High yield	Low yield
Mixing	Difficult mixing	Easy mixing
Process control	Difficult to control pH, temperature, and oxygen	Easy to control pH, temperature, and oxygen
Extraction	Easy extraction	Complex extraction
Scalability	Small-scale manufacturing	Large-scale manufacturing
Uniformity	Provide a less homogenous environment.	Provide more homogenous environment.
Cost-effectiveness	Use substrates with relatively lower cost, such as agricultural residues.	Use substrates with relatively higher cost.
Example	β-carotene from *R. rubra*	β-carotene from *E. acetylicum* S01

## Step IV: extraction of carotenoids from bacteria

Carotenoids extraction is challenging, as they are intracellular molecules that are rarely soluble in solvents and are extremely vulnerable to oxidative destruction [27]. To extract carotenoids from the microbial biomass, two stages are used: first, the microbial cell wall must be disrupted, and then the carotenoid must be dissolved in an organic solvent [154]. There are three ways to disturb cells: chemically, physically, and biologically. Physical methods include the use of a mortar and pestle, vortex mixing with or without glass beads, orbital shaking, and incubation (which may involve a temperature rise). Chemical techniques employ acids, bases, or surfactants, whereas biological techniques employ enzymes like cellulase, pectinase, or lysozyme to disrupt cells [29]. After that, the carotenoids must be extracted from the cell slurry and other metabolites [27]. Many organic solvents have been utilized (singly or in combination) to extract bacterial carotenoids either concurrently or after cell destruction, including hexane, acetone, ethyl acetate, dichloromethane, dimethyl sulfoxide, methanol, and ethanol [155]. Selecting the extraction solvent may be simpler for bacteria that only produce carotenes, such as β-carotene, lycopene, etc. However, the solvent selection phase becomes more complex when bacteria produce xanthophylls and carotenes simultaneously [84]. Another important factor to take into account during carotenoid extraction is the cell wall of the bacteria. For example, Gram-negative bacteria contain lipopolysaccharide in the outer membrane and a thin peptidoglycan layer separating their inner and outer membranes, which makes them more permeable to organic solvents. However, Gram-positive bacteria are more resistant to organic solvents because they have a strong cell wall made mostly of peptidoglycan, which covers their inner membrane [156]. Although most studies support the effectiveness of organic solvents as a preliminary step in the extraction of carotenoids from microbial cells, the toxicity of their byproducts still poses a risk to human health. Therefore, it is necessary to look into safer and greener alternatives, especially when the final product is intended for a highly valuable formulation (Table 6) [157].‏

**Table 6:** Greener alternatives for the extraction of microbial carotenoids

Extraction technique	Mechanism of action	Example	Characteristics	References
Microwave-assisted extraction	Uses microwave energy to break down the cell membrane, releasing carotenoids into the solvent as a result.	Extraction of astaxanthin from *P. carotinifaciens*.	Advantages: Minimal time and solvents, great yield rate, and relatively cheap. Disadvantages: Expensive equipment and clean up are required.	[11, 27, 158, 159]
Enzymatic extraction	Proteases, lipase, cellulase, lysozyme, or enzyme mixtures are employed for microbial cell lysis. For instance, the β-1-4-glucosidic bonds in peptidoglycan are hydrolyzed by lysozyme application, which also breaks down the membranes of bacteria.	Extraction of β-carotene from *D. salina* using lipase.	Advantages: Mild environment, high specificity, and superior efficiency. Disadvantages: High cost and susceptibility to deterioration.	[11, 23, 27, 160]
Ionic liquids and deep eutectic solvents	Ionic liquids are low-melting-point salts, while deep eutectic fluids are binary or ternary combinations of substances that have melting points much less than those of their individual components. These fluids break down cells and make it easier to retrieve intracellular substances.	Extraction of carotenoid from *G. alkanivorans* strain 1B using both imidazolium- and phosphonium-based ionic liquids.	Advantages: High thermal and chemical stability and great solvent power. Disadvantages: Toxicity and high cost.	[27, 161]
Supercritical fluid extraction	Disperses through a solid’s pores like a gas and has the ability to dissolve substances like a liquid. These characteristics make supercritical fluids appropriate solvents for the extraction of delicate substances like carotenoids.	Extraction of β-carotene from *Rhodosporidium paludigenum* using carbon dioxide	Advantages: High safety and quick carotenoid yields. Disadvantages: High cost.	[11, 23, 159, 162]
Ultrasonic-assisted extraction	Generates high-frequency ultrasonic vibrations that allow the solvent to enter the cell deeply and sufficiently homogenize the solvent with the pigment.	Extraction of flexirubin from *Chryseobacterium artocarpi* CECT 8497.	Advantages: Cost-effective and high extraction performance. Disadvantages: Deterioration of compounds.	[11, 23, 159]
Natural solvent mixtures	Mixtures of natural solvents made mostly of natural ingredients, like menthol, bio-based alcohols, and bio-based carboxylic acids, can be believed to be viable substitutes for traditional solvents.	Extraction of astaxanthin from *P. carotinifaciens* by employing menthol combined with ethyl acetate and ethanol.	Advantages: Consumer preference and high safety. Disadvantages: Limited solvent power.	[163]
Microemulsion technique	This technique uses surface-acting chemicals called surfactants, which can reduce molecular surface tension even in trace amounts, altering the polarity of hydrophobic substances and improving extraction.	Extraction of β-carotene using short-chain fatty acids in aqueous media.	Advantages: Specificity. Disadvantages: Toxicity and high cost.	[159, 164]

### Biological application of bacterial carotenoids

Numerous health benefits have been linked to carotenoids; their circulating levels and dietary intake have been linked to lower rates of diabetes, several cancers, and even lower overall mortality [57]. The carotenoids that are isolated from bacteria are just as safe for human consumption as those that come from more conventional sources like plants or synthetic chemicals [82]. Besides their low toxicity, bacterial carotenoids are commonly used due to their antioxidant property, stability in acidic environments, and neutral pH, as well as their strong coloring power [75]. The therapeutic applications of bacterial carotenoids are listed in Table 7.

#### Bacterial carotenoid as an antibacterial agent

**Fig. 8:**
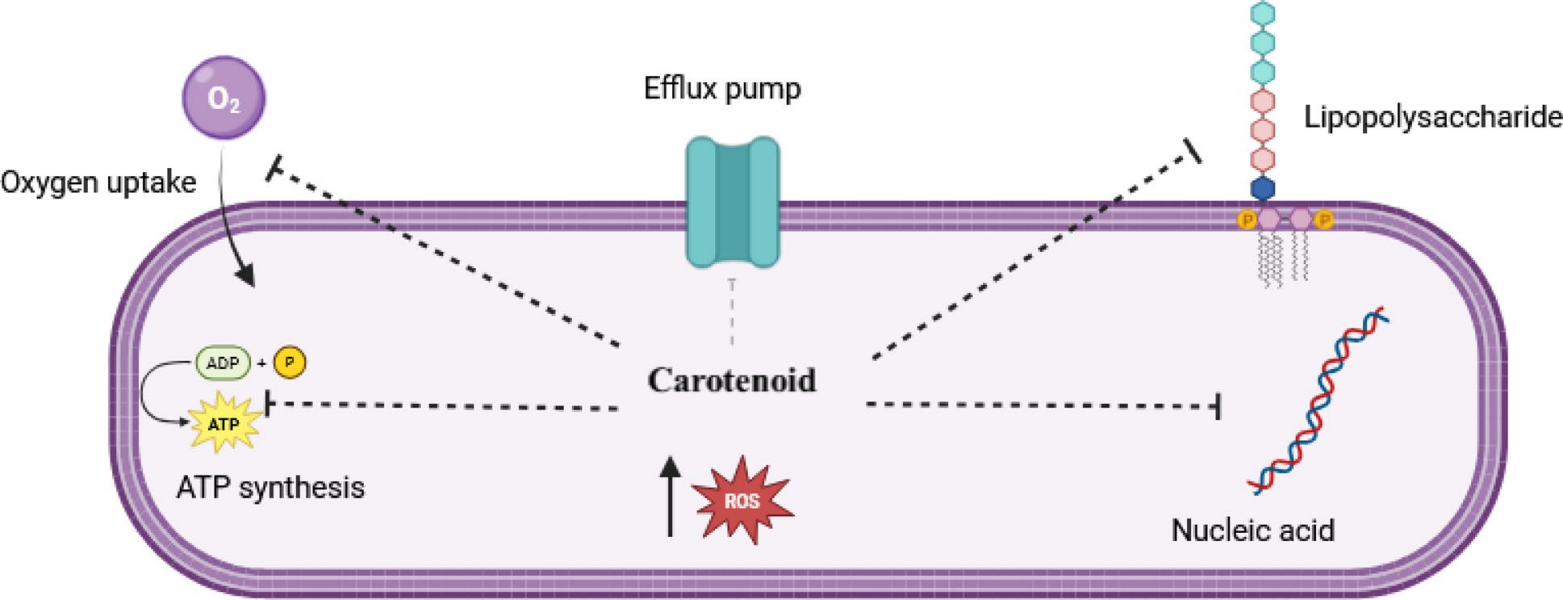
The carotenoids exert their antibacterial action via damaging lipopolysaccharide (LPS) and nucleic acid, preventing ATP formation and oxygen uptake, disrupting the efflux pump, and increasing ROS formation inside the bacterial cell

The antibacterial properties of bacterial pigment, particularly carotenoids, have been documented in numerous studies against various bacterial species (Fig. 8) [165]. Ibrahim [50] uncovered the antibacterial activities of carotenoids of *H. scabra*, *Holothuria leucospilota*, and *Holothuria atra* against a number of human and fish pathogens, such as *S. aureus* ATCC 6538, *Pseudomonas aeruginosa* ATCC 8739, *Vibrio damsela*, *Streptococcus faecalis*, and *E. coli*. Further, the carotenoid pigments of *M. roseus* and *M. luteus* exhibited antibacterial activity against some human pathogens, such as *E. coli*, *S. aureus*, and *S. faecalis* [166]. According to Boontosaeng et al. [167], carotenoid pigments from *Bacillus* sp., *Corynebacterium* sp., *Kocuria roseus*, *Staphylococcus* sp., and *Brevibacterium* sp. exhibited antibacterial activity against *S. aureus* ATCC 25923, *Staphylococcus* xylosus, *Bacillus macerans*, *Citrobacter diversus*, and *Aeromonas schubertii*. In addition, Rostami et al. [51] found that the carotenoid pigments produced by *M. roseus* and *Rhodotorula glutinis* showed antibacterial activity against all tested bacteria, such as *Bacillus cereus*, *Streptococcus pyogenes*, *E. coli*, *Salmonella enteritidis*, *Enterococcus faecalis*, and *Listeria monocytogenes*. Moreover, Alshamaa and Issam [168] discovered that the carotenoid pigment staphyloxanthin had the highest antibacterial activity against *Staphylococcus epidermidis*, *Acinetobacter baumannii*, and *Proteus mirabilis*, and the lowest activity appeared against both *P. aeruginosa* and *Shigella dysenteriae*. Furthermore, the carotenoid pigment produced by *Staphylococcus gallinarum* KX912244 presented considerable antibacterial properties against *S. aureus* and *E. coli* [169]. Additionally, carotenoids of *Flavobacterium* sp. and *Brevibacterium* sp. exhibited good antibacterial activity with maximum effect on *E. coli*, *Corynebacterium diphtheriae*, and *S. aureus* [56]. According to Sidin and Retnaningrum [170], the carotenoid pigment of *Kocuria rhizophilla*, *Calidifontibacter* sp., and *Rhodococcus ruber* showed antibacterial properties against *E. coli* and *S. aureus*. Besides, the carotenoid pigment from *Micrococcus lylae* MW407006 showed antimicrobial effect against different multi-drug-resistant (MDR) *Klebsiella pneumoniae*, *E. coli*, *Proteus vulgaris*, *P. aeruginosa*, *Salmonella typhi*, *L. monocytogenes*, *(A) baumannii*, and *S. aureus* [128]. The crude extract of the carotenoid showed the extreme antibacterial action against *Yersinia enterocolitica* ATCC 27,729, followed by *S. aureus* NCTC 10,788, *E. coli* ATCC BAA-2523, *Streptococcus salivarius* ATCC 13,419, and *(B) cereus* BC 6830 [171]. The study conducted by Kusmita et al. [172] showed that the carotenoid pigment produced by *Virgibacillus* sp. exerted outstanding antibacterial activity against methicillin-resistant *S. aureus* (MRSA) and MDR *E. coli*. Finally, Hagaggi and Abdul-Raouf [59] reported that the β-carotene pigment from *(C) parietis* AUCs exerted a promising antibacterial activity against *S. aureus* ATCC 25923, *Streptococcus agalactiae* ATCC 13813, *P. aeruginosa* ATCC 9027, and *K. pneumoniae* ATCC. 4352 with variable inhibition zone diameters according to bacterial species.

#### Bacterial carotenoid as an antifungal agent

Rostami et al. [51] found that the carotenoid pigment inhibited the fungal growth of *Penicillium digitatum*. Further, the *M. luteus*’s carotenoid pigment showed a promising antifungal activity against *Aspergillus niger*, *Alternaria* sp., *Aspergillus terreus*, *Curvularia*, *Cladosporium* sp., and *Penicillium certum* [173]. Furthermore, carotenoid pigment extracted from *S. gallinarum* KX912244 showed antifungal potential against *Candida albicans* [169]. Moreover, a yellow-colored carotenoid pigment extracted from *Bacillus gibsonii* exhibited selective antifungal action against diverse fungi, namely *Rhizoctonia solani* and *Sclerotium rolfsii* [174]. Additionally, Shahin et al. [128] reported the antifungal activity of the carotenoid pigment produced by *M. lylae* MW407006 against *C. albicans* using the disc-diffusion method.

#### Bacterial carotenoid as an antibiofilm agent

Majeed [173] reported the antibiofilm activity of carotenoid pigment by reporting that all biofilm-producing isolates of *E. coli*, *B. subtilis*, *S. aureus*, *K. pneumoniae*, and *P. aeruginosa* became non-producers after application of carotenoid. Furthermore, *P. aeruginosa*’s biofilm was reduced by 50% in the presence of 2–4 µg/ml of the *Rhodococcus* sp. SC1’s carotenoid pigment, while this pigment led to a 50% reduction of *E. coli*’s biofilm at a concentration of 0.5 µg/ml, and no biofilm formation was observed at a concentration less than 64 µg/ml [175].

#### Bacterial carotenoid as an anticancer agent

**Fig. 9:**
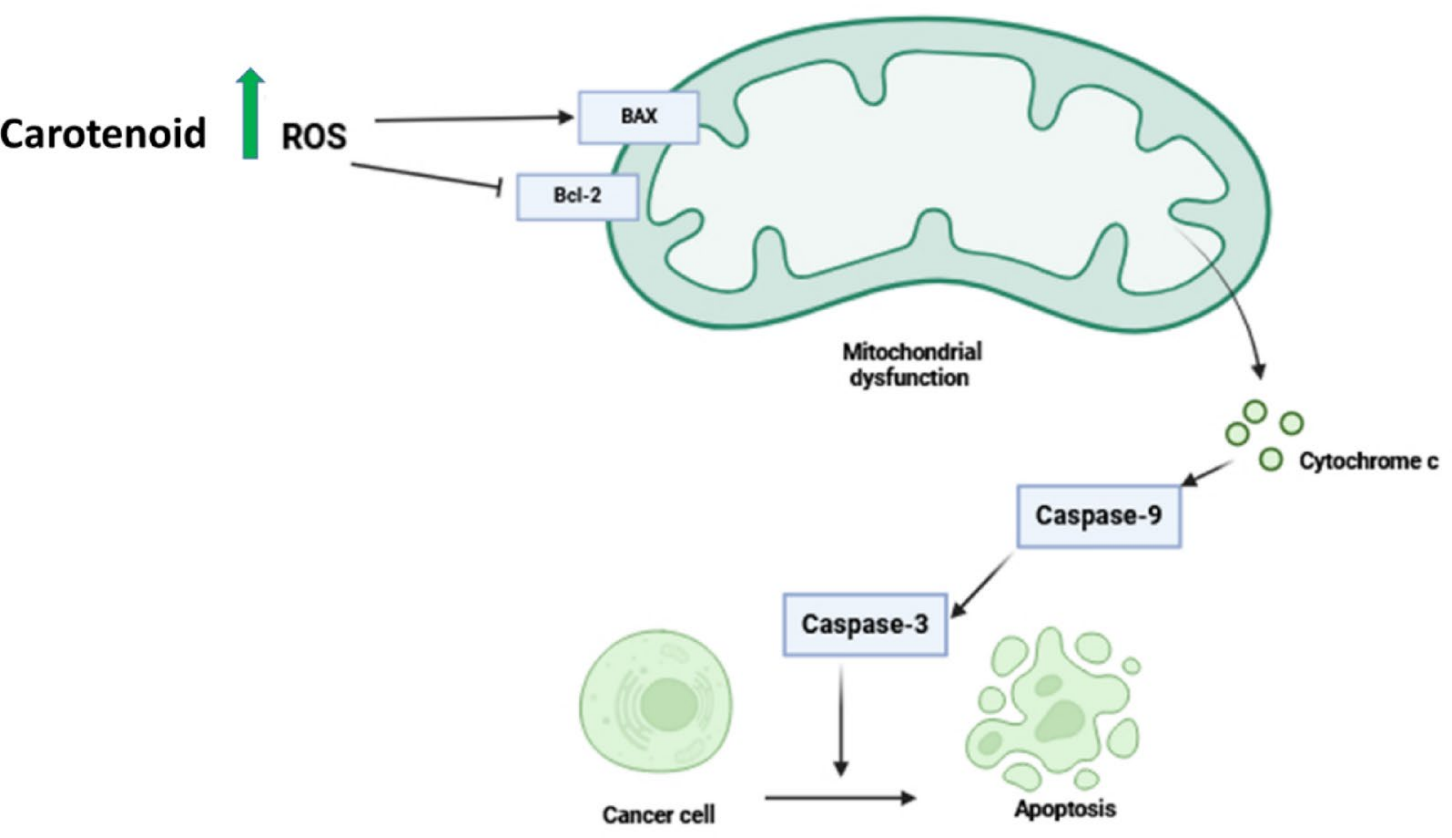
Carotenoids induce ROS production, which upregulates the BAX gene and downregulates the Bcl-2 gene, causing mitochondrial impairment that provokes cytochrome c release and activates caspase proteins that lead to apoptosis

Numerous in vitro and in vivo studies have demonstrated the carotenoids’ anticancer capabilities, and various authors have reported the anticancerous properties of lutein, fucoxanthin, astaxanthin, zeaxanthin, and β-carotene (Fig. 9) [176]. As stated by Rostami et al. [51], by using a two-stage carcinogenesis method for mouse skin papillomas induced by TPA, the carotenoid pigments produced by *M. roseus* and *R. glutinis* demonstrated in vivo antitumor-promoting activities, as they significantly inhibited the formation of papillomas and decreased the number of papillomas per mouse. In addition, the extracted carotenoid from *Kocuria* sp. strain QWT-12 exhibited anticancer activity against seven different cancer cell lines belonging to breast cancer (MDA-MB-468, MDAMB-231, and MCF-7), lung cancer (A549), and prostate cancer (DU145, PC3, and LNCaP), with IC_50_ values ranging from 1 to 8 mg/ml [89]. According to Barretto and Vootla [169], staphyloxanthin carotenoid showed anticancer action against four dissimilar cancer cell lines like the Dalton’s lymphoma ascites, Ehrlich ascites carcinoma, A549 lung carcinoma, and skin melanoma (B16F10) with IC_50_ value 6.20 ± 0.02, 6.48 ± 0.15, 7.23 ± 0.11, and 6.58 ± 0.38 µg/ml, respectively. Additionally, the carotenoids isolated from *E. acetylicum* S01 demonstrated a significant dose-dependent inhibition of the viability of colorectal cancer HT-29 cells [54]. Furthermore, carotenoids obtained from *Deinococcus* sp. UDEC-P1 reduced the viability of human osteosarcoma Saos-2 cells (37.1%), while the carotenoid from *Arthrobacter* sp. UDEC-A13 showed a significant inhibition of the viability of neuroblastoma Neuro-2a cells (20.6%), human osteosarcoma Saos-2 cells (26.3%), and breast cancer MCF-7 cells (13.2%) [177]. Moreover, *M. luteus*’s carotenoid pigment revealed mild to severe cytotoxicity to HeLa cells with An IC_50_ value of 36.09 ± 0.216 µg/ml [91].

#### Bacterial carotenoid as an antioxidant agent

Antarctic microorganism from the *Pedobacter* genus generates different carotenoids, which have a strong antioxidant capacity that was measured by three different methods, including DPPH, ROS detection, and oxygen electrode [178]. The carotenoid that was isolated from *Kocuria marina* DAGII demonstrated 76.88% radical scavenging activity, with an IC_50_ value of 53.76 µg/ml [179]. According to Barretto and Vootla [169], the carotenoid pigment generated by *S. gallinarum* KX912244 exhibited in vitro antioxidant activity, measured by percentage DPPH free radical scavenging activity, with an IC_50_ value of 54.22 µg/ml. In addition, the carotenoid extracted from *Lactobacillus plantarum* subsp. *Plantarum* has promising antioxidant activity [180]. Additionally, *Planococcus* sp. ANT_H30 and *Rhodococcus* sp. ANT_H53B were proven to synthesize crude carotenoid extracts that demonstrated a notable capacity to scavenge free radicals [181]. Besides, the yellow pigment derived from *M. luteus* demonstrated 29.6 ± 0.32% free radical scavenging activity [182]. In addition, DPPH and ABTS assays were used to assess the antioxidant ability of the new carotenoid from *Metabacillus idriensis* LipT27 to scavenge free radicals, and the amount of pigment needed for ABTS IC_50_ was 72 ± 3 µg/ml, while for DPPH IC_50_ was 26 ± 2 µg/ml [171]. Further, according to Kusmita et al. [172], the carotenoids isolated from *Virgibacillus* sp. showed substantial antioxidant activity using the DPPH method. Moreover, β-carotene generated by *C. parietis* AUCs had significant antioxidant activity with DPPH scavenging action equal to 87% [59]. Finally, the DPPH assay revealed that the crude carotenoid pigment extracted from endophytic *M. luteus* exhibited a notable dose-dependent inhibition with an IC_50_ value of 2.29 µg/ml [91].

#### Bacterial carotenoid as an anti-inflammatory agent

**Fig. 10:**
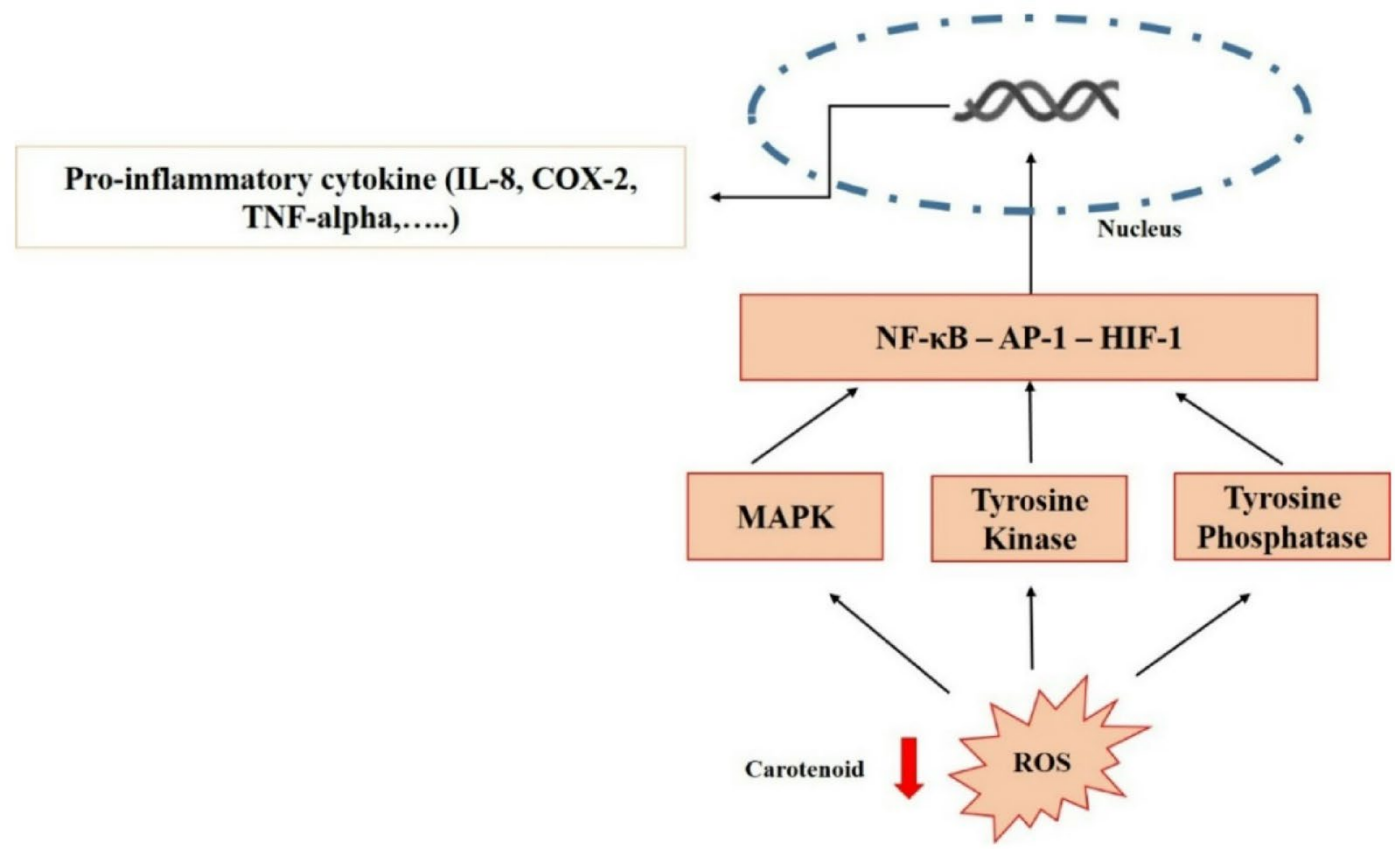
The ROS-mediated production of pro-inflammatory cytokines is inhibited by carotenoids via a reduction in the formation of ROS

As stated by Peerapornpisal et al. [183], carotenoids help to control chronic inflammatory illnesses by significantly reducing the production of prostaglandins, nitric oxide (NO), and pro-inflammatory cytokines (Fig. 10). According to Rostami et al. [51], when TPA-induced inflammation in mice was tested, the carotenoid pigments made by *R. glutinis* and *M. roseus* were found to have significant anti-inflammatory effects, with ID_50_ values of 0.09 and 0.22 mg/ear, respectively. As per Lopes et al. [53], the anti-inflammatory properties of the carotenoid pigment generated by *N. antarctica* LEGE13457 were assessed by observing a decrease in NO generation by RAW 264.7 cells following lipopolysaccharide stimulation, with an IC_25_ of 22.2 ± 1.6 µg/ml. Moreover, the photosynthetic bacteria *R. sphaeroides* (WL-APD911) were found to create the carotenoid extract “LycogenTM,” which has the potential to be a bioactive mixture with anti-inflammatory and anti-oxidative characteristics [72]. Furthermore, *E. acetylicum* S01’s carotenoids significantly reduced lipid peroxidation, TNF-α, and NO generation in PBMCs stimulated by lipopolysaccharide [54].

#### Bacterial carotenoid as an antidiabetic agent

**Fig. 11:**
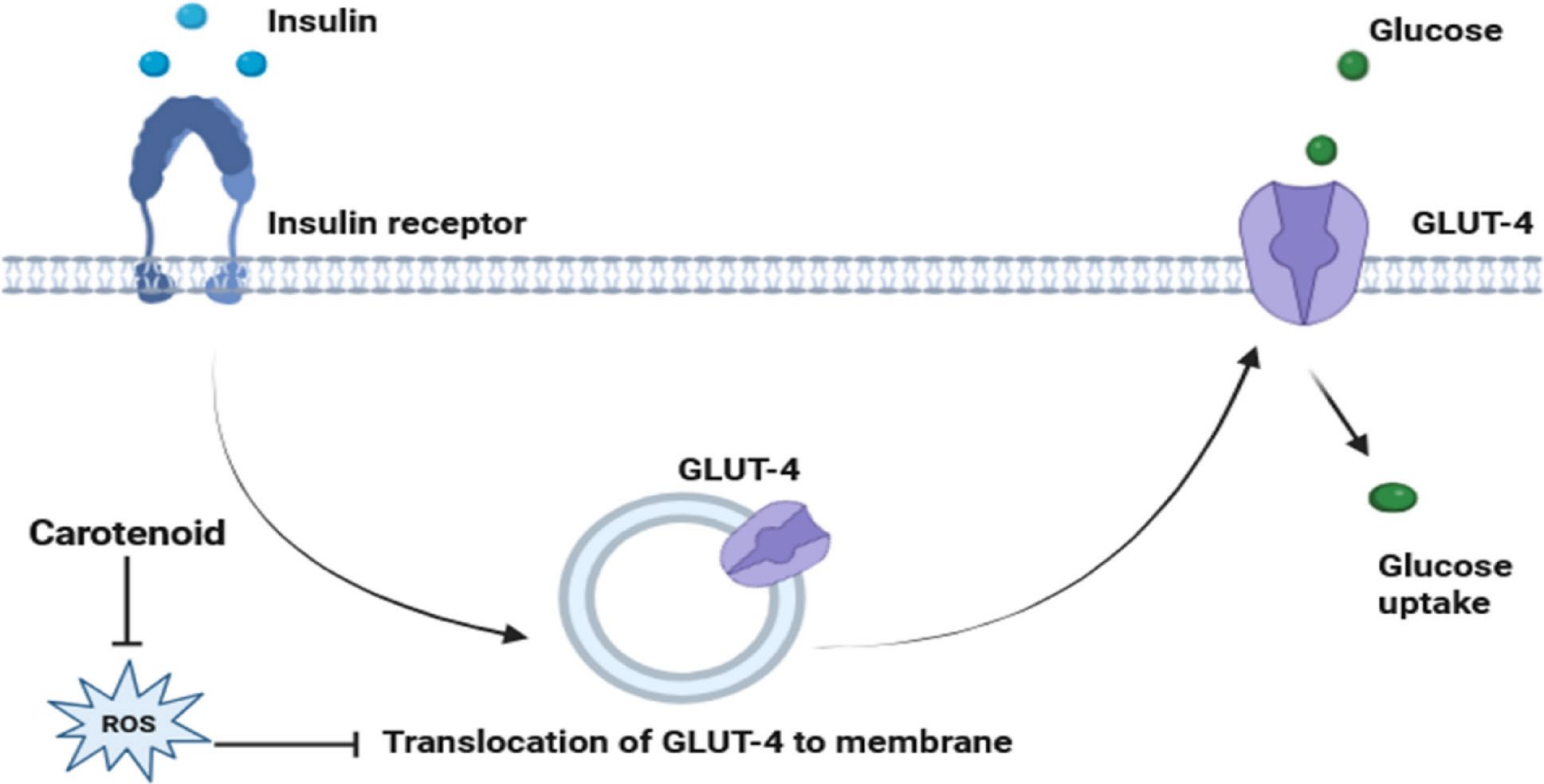
Binding of insulin to its receptors activates glucose transporter type 4 (GLUT4) translocation, which leads to glucose uptake. In the case of a high level of ROS, GLUT4 translocation and glucose uptake were impaired. Carotenoids exert their antidiabetic action through lowering ROS formation and hence preventing diabetic complications

Carotenoids have been shown to have promise in the treatment and management of diabetes, according to recent studies. It has been demonstrated that carotenoids reduce the risk of type 2 diabetes mellitus in humans and that there is a negative correlation between carotenoids and glucose levels [184]. It was reported that oxidative stress is associated with many diseases, such as obesity and diabetes, and by scavenging these fatty acid radicals and ROS with carotenoids, these diseases could be reduced by 40 to 79% (Fig. 11) [185]. Sayahi and Shirali [186] suggested that lutein, lycopene, β-carotene, and astaxanthin have the potential to defend against diabetes. According to Hagaggi and Abdul-Raouf [59], β-carotene exhibited antidiabetic properties by enhancing the yeast cells’ absorption of glucose in a proportionate way to the glucose concentration, meaning that the uptake percentage rose as the glucose concentration increased. Moreover, it has been discovered that β-carotene enhances lipid formation and glucose metabolism. Utilization in muscle and liver cells to produce its hypoglycemic effect [187].

#### Bacterial carotenoid as a food additive

Due to their antioxidant properties and their role as precursors of vitamin A, carotenoids are essential as food additives and not just as natural colorants [188]. In accordance with Rowles et al. [71], zeaxanthin derived from bacterial sources has the potential to reduce the risk of human carcinogenesis, and the World Health Organization and the United Nations have established a recommended daily consumption of zeaxanthin per kilogram of body weight, which is ≤ 2 mg/kg. Furthermore, astaxanthin (E161j, food dye approved by the European Commission) is utilized as a nutritional supplement and food colorant in the form of oils and tablets [29]. In addition, astaxanthin generated by *M. lacticola* is utilized in fish nutrition because of its antioxidant properties and ability to provide a red tint that appeals to customers [82], while *P. carotinifaciens*’s astaxanthin is used in the coloration of rainbow trout and salmon [189]. According to Manikandan et al. [190], canthaxanthin pigment is used in the pigmentation of salmon flesh. Pasarin et al. [191] reported that the addition of zeaxanthin derived from *Flavobacterium* sp. to poultry feed resulted in a deeper yellow hue for both the skin and yolks of chickens.

**Table 7:** The therapeutic applications of bacterial carotenoids

Bacterial source	Carotenoid	Application	Target	Dose	Mechanism	Reference
*H. scabra*	Xanthophyll/β-crptoxanthin/β-carotene	Antibacterial	*S. aureus* ATCC 6538	1.4 µg/ml	NI	[50]
*C. parietis* AUCs	β-carotene	Antibacterial	*S. aureus* ATCC 25923/*S. agalactiae* ATCC 13813/*P. aeruginosa* ATCC 9027/*K. pneumoniae* ATCC 4352	1 mg/ml	Membrane damage	[59]
		Antioxidant	DPPH assay		Scavenge free radicals	
		Antidiabetic			Inhibit pancreatic α-amylase activity / Enhancing the absorption of glucose	
*Rhodococcus* sp. SC1’s	NI	Antibacterial	*P. aeruginosa* *E. coli*	32 µg/ml 64 µg/ml	NI	[175]
*M. luteus*	NI	Antibiofilm	*E. coli*/*B. subtilis*/*S. aureus*/*K. pneumoniae*/*P. aeruginosa*	1 mg/ml	NI	[173]
*S. gallinarum* KX912244	Staphyloxanthin	Antibacterial	*S. aureus*/*E. coli*	50, 75, and 100 µg/ml	NI	[169]
		Antifungal	*C. albicans*		NI	
		Anticancer	Dalton’s lymphoma ascites/Ehrlich ascites carcinoma / A549 Lung carcinoma/skin melanoma (B16F10)	IC_50_ = 6.20, 6.48, 7.23, and 6.58 µg/ml	NI	
		Antioxidant	DPPH assay	IC_50_ = 54.22 µg/ml	Scavenge free radicals	
*L. plantarum* subsp. *plantarum*	4, 4′-diaponeurosporene	Antioxidant	DPPH assay	NI	Scavenge free radicals	[180]
*Rhodococcus* sp. *ANT_H53B*	dihydroxyneurosporene and hydroxyechinenone	Antioxidant	DPPH assay	IC_50_ = 0.96 µg/ml	Scavenge free radicals	[181]
*M. luteus*	NI	Antioxidant	RAP/ABTS/DPPH assays	50 µg/ml, 500 µg/ml, and 1000 µg/ml	Scavenge free radicals	[182]
*K. rhizophilla*	Zeaxanthin	Antibacterial	*E. coli*/*S. aureus*.	0.16 mg/ml	NI	[170]
*Micrococcus* spp.	NI	Antibacterial	*E. coli*/*S. aureus*/*Streptococcus faecalis*	0.25-10 mg/ml	NI	[166]
*Corynebacterium* sp.	NI	Antibacterial	*S. aureus ATCC 25923 (MSSA)/ S. xylosus/ B. macerans/ Citrobacter divesus/A. schubertii*	NI	NI	[167]
*S. aureus*	Staphyloxanthin	Antibacterial	*S. epidermidis*/*A. baumannii*/*P. mirabilis/P. aeruginosa*/*S. dysenteriae*	NI	NI	[168]
*Flavobacterium* genus	Zeaxanthin	Antibacterial	*E. coli*/*K. pneumoniae*/*P. aeruginosa*/*S. aureus*/*B. subtilis*/*Sarcina lutea*/*C. diphtheriae*	0.5-5 mg/ml	NI	[56]
*M. lylae* MW407006	Echinenone	Antibacterial	*P. aeruginosa/S. aureus*	256.0 µg/ml / 128.0 µg/ml	NI	[128]
		Antifungal	*C. albicans*	512 µg/ml	NI	
*B. gibsonii*	NI	Antifungal	*R. solani/S. rolfsii*	NI	NI	[174]
*M. idriensis* LipT27	NI	Antibacterial	*Y. enterocolitica/S. aureus/E. coli*	100 and 250 µg/ml	NI	[171]
		Antioxidant	DPPH/ABTS	IC_50_ = 26 ± 2 µg/ml/IC_50_ = 72 ± 3 µg/ml	Scavenge free radicals	
*Virgibacillus* sp.	NI	Antibacterial	Methicillin-resistant *S. aureus*/*E. coli*	NI	NI	[172]
		Antioxidant	DPPH	IC_50_ = 506 ppm	Scavenge free radicals	
*M. luteus*	NI	Anticancer	HeLa cancer cell line	5 to 100 µg/ml	NI	[91]
		Antioxidant	DPPH assay	IC_50_ = 2.29 µg/ml	Scavenge free radicals	
*Kocuria* sp. strain QWT-12.	Neurosporene	Anticancer	Breast cancer (MDA-MB-468, MDAMB-231, and MCF-7)/Lung cancer (A549)/Prostate cancer (DU145, PC3, and LNCaP)	IC_50_ ranged from 1 to 8 mg/ml	NI	[89]
*Deinococcus* sp. UDEC-P1	Deinoxanthin	Anticancer	Human osteosarcoma Saos-2 cell	40 µg/ml	NI	[177]
*E. acetylicum* S01	Diapolycopenedioic-acid-diglucosyl ester/keto-myxocoxanthin glucoside ester	Anticancer	Colorectal cancer HT-29 cells	25 to 150 µM	NI	[54]
		Anti-inflammatory	PBMCs stimulated by lipopolysaccharide	NI	Reduced lipid peroxidation, TNF-α, and nitric oxide generation.	
*Pedobacter* genus	NI	Antioxidant	DPPH/Oxygen electrode	NI	Scavenge free radicals	[178]
*K. marina* DAGII	NI	Antioxidant	DPPH	IC_50_ = 53.76 µg/ml	Scavenge free radicals	[179]
*N. antarctica* LEGE13457	β-carotene/Zeaxanthin/Echinenone/Lutein	Anti-inflammatory	LPS-stimulated inflammation in RAW 264.7 cells	IC_25_ = 22.2 ± 1.6 µg/ml	Decrease in nitric oxide generation	[53]
*R. glutinis*	NI	Anti-inflammatory	TPA-induced inflammation in mice	ID_50_ = 0.09 mg/ear	NI	[51]
*M. lacticola*	Astaxanthin	Food additive	Fish nutrition	NI	Antioxidant properties and the ability to provide a red tint that appeals to customers	[82]
*P. carotinifaciens*	Astaxanthin	Food additive	Coloration of rainbow trout and salmon	NI	Ability to provide a tint	[189]
*Flavobacterium* sp.	Zeaxanthin	Food additive	Poultry feed results in a deeper yellow hue for both the skin and yolks of chickens	NI	Ability to provide a tint	[191]

NI: Not identified.

## Limitations and future perspectives

Despite the privileges of bacterial carotenoids, there are some challenges that limit their applications, concerning high production cost, low productivity of the rare carotenoids, difficult extraction, and low bioavailability and stability. These limitations and their possible tackles are discussed in this section.

Limitation (1): Despite the advantages of bacterial carotenoids, the market share of these carotenoids is limited due to high production costs, compared to chemical synthesis. The following may lower the cost of production;

- Screening of highly productive strains: Choosing of new strain that grows quickly and accumulates a high amount of carotenoids leads to a high carotenoid yield and will reduce production cost, as only a few bacterial sources are known. For rapid screening of a highly productive strain, the utilization of an electrochemical sensor will be beneficial, which provides a repeatable and high-performance analysis for rapid screening of the natural pigment production [192]. For instance, the reduced graphene oxide and methionine film modified screen-printed carbon electrode for evaluating food colorants, such as tartrazine, carminic acid, and amaranth [192]. Two phases are involved in this process: drop-casting reduced graphene oxide and electropolymerizing of poly (L-methionine) film over a screen-printed carbon electrode. After that, cyclic voltammetry and electrochemical impedance spectroscopy were used to examine each dye’s electrochemical behavior [192].
- Co-culturing: To increase biosynthesis efficiency, scientists have started using several heterogeneous organisms that grow together in a single fermentation [193]. Because the co-culture approach activates under-expressed pathways and produces novel metabolites [194], these co-culture investigations have shown feats of chemical biosynthesis not previously accomplished in a single strain [193]. Co-culturing *P. haeundaensis* with *Lactobacillus fermentum* increased the synthesis of astaxanthin by 2.5 times [195]. Additionally, lutein and zeaxanthin productivity increased by 3.54 and 4.81 times, respectively, when *Chlorella saccharophila* (UTEX247) was co-cultured with *Exiguobacterium* sp. strain AMK1 in nitrate-depleted conditions and supplemented with 3% CO₂ [194].
- Low-cost substrate: The development of low-cost pigment synthesis using low-cost substrate is necessary due to the high cost of present bioproduction techniques [196]. For example, *P. anthophila* FL1_IS5 produced more carotenoids when carrot pomace hydrolysate was added as a co-substrate in the cultivation [106]. Jiang et al. (2023) reported that *Rhodococcus aetherivorans* N1 could directly synthesize 3 mg of carotenoids, including zeaxanthin and β-carotene, from the undetoxified lignocellulosic hydrolysate [197]. Furthermore, using rice powder as the only substrate, *Bacillus clausii* produced a maximum yield of carotenoid pigment of 95.8 mg/3 g rice powder at ideal experimental conditions (pH = 7, temperature 30 °C, and moisture 55%) [196]. Further, the production of carotenoid by *M. luteus* was improved by supplementation the medium with 3% whey [129]. In addition, the production of β-Carotene in the *Pantoea dispersa* MSC14 strain has been boosted by 13.97% by adding maize steep liquor powder to the culture medium [198].

Limitation (2): Some bacterial sources produce rare carotenoids with extremely low yield, and there is an urgent need to improve the production of these carotenoids due to their medical applications. The yield of these rare carotenoids was augmented as follows;

- Molecular chaperones: For the majority of proteins to become functionally active, they must fold into certain three-dimensional structures. However, newly synthesized proteins are extremely vulnerable to abnormal folding in the cellular environment, which could result in the formation of hazardous species [199]. Cells invest in a sophisticated network of molecular chaperones to prevent these threats, facilitating effective folding [199]. These molecular chaperones can be used to increase the activity of carotenogenic enzymes [200]. For instance, employing a commercial molecular chaperone of pG-KJE8 increased the soluble production and catalytic activity of CrtW enzyme, a crucial enzyme in the production of canthaxanthin, thereby increasing the production of canthaxanthin in *E. coli* by 1.16 times [200].
- Microfluidics: The lack of systematic strain optimization is holding back progress towards industrialization. Microfluidics provides a strong way to adjust culture medium composition, allowing for high-throughput screening and precise control over nutrient concentrations, which leads to increased biomass output and yield development. Researchers can use microscale channels and droplet-based devices to explore a large parameter space of medium formulations with low reagent usage and minimal experiment time [201]. For example, nitrate and phosphate were the critical factors in cyanobacterial cultivation medium, as demonstrated by the high-throughput optimization of cyanobacterial cultivation utilizing droplet-based microfluidics [201].
- Clustered regularly interspaced short palindromic repeats (CRISPR): Recently, bioactive components have been produced on a large scale using genetic engineering techniques utilizing CRISPR-based genome editing [202]. For instance, the ZEP2 and ZEP3 genes were knocked out of *Phaeodactylum tricornutum* using CRISPR/Cas9 technology, allowing for the industrial synthesis of the bioactive pigment diatoxanthin [203]. Furthermore, the *B. subtilis* strain was genetically engineered with CRISPRi to generate a stable, safe, and effective C30 carotenoid [90].
- Self-assembled protein nanocages: In biotechnology domains like natural product biosynthesis, the development of self-assembled protein nanocages for enzyme immobilization has great promise [204]. As an example, using a constructed protein cage based on the α-carboxysome, the enzymes involved in IPP biosynthesis (CrtE/CrtB/CrtI) were integrated onto the outside, increasing lycopene production in the engineered *E. coli* by 1.7 times when compared to the control strain [204].
- Slot 20 assignment cloning (SA-Clo): A new approach known as SA-Clo, which uses the modular cloning method, was presented to enable the specific production of different carotenoids by facilitating the quick assembly of operons with different constructs [205]. In *E. coli*, SA-Clo offers the ability to produce plasmids for a range of minor and uncommon carotenoids, including violaxanthin, 4,4′-diapophytoene, 4,4′-diaponeurosporene, and 4,4′-diapolycopene [205].
- Artificial intelligence (AI)-driven enzyme: AI can support the development of enzyme engineering with customized and optimized functionality. The production of rare carotenoids can be improved by optimizing enzyme performance using AI-driven enzyme design, which reduces the time and cost associated with the industrial manufacturing of enhanced enzymes [206]. It has been shown that numerous generative AI models can create enzymes that do the same tasks as their natural counterparts, such as lactate dehydrogenases, luciferases, lysozymes, recombinases, and proteases [207].

Limitation (3): The poor solubility and lipophilicity of carotenoids severely limit their intestinal absorption and biological effectiveness. Furthermore, their polyene backbone is extremely prone to deterioration in a variety of environmental circumstances, such as high temperatures, light exposure, oxidative stress, and acidic pH [208]. To overcome these challenges, several strategies have been proposed to enhance the stability and bioavailability of carotenoids as follows;

- Emulsion formation: For intestinal epithelial cells to absorb carotenoids, they must be emulsified by dietary lipids and then incorporated into bile salt micelles. When fat digestion or micelle production is disrupted, carotenoid bioavailability is dramatically reduced [209]. Dissolving carotenoids in an oil phase to create an emulsion has been shown to be a successful method for addressing their poor water solubility [209]. For example, in soybean O/W microemulsions, the bioaccessibility of β-carotene and lycopene rose to 17.3% and 63.8%, respectively, whereas in sunflower O/W microemulsions, it rose to 17.8% and 71.1%, respectively [210].
- Microencapsulation and liposome: Microencapsulation and liposome encapsulation technologies are among the most promising strategies. By forming protective walls around carotenoid molecules, these encapsulation systems safeguard them from harmful environmental factors, such as heat, light, oxidation, and acidic pH. Consequently, carotenoids’ gastrointestinal absorption and chemical stability are significantly enhanced [211]. The probiotic bacteria *Lactobacillus nososus* GG were spray-dried with β-carotene-liposomes, resulting in biphasic dried microparticles that demonstrated high storage durability for 90 days at room temperature [212]. Furthermore, the β-Cryptoxanthin-encapsulated chitosome could be stored at 4 °C for up to 30 days and had a half-life of 116 days with a degradation rate constant of 0.006/day [213].
- Nanotechnology: Delivery systems based on nanotechnology have become a potent technique in recent years to overcome the stability and solubility problems of carotenoids. Improved water dispersibility, increased bioavailability, controlled release, and targeted distribution are just a few benefits of encapsulating carotenoids within nanoparticles, such as polymeric nanoparticles, nanoliposomes, or nanoemulsions [214]. For example, astaxanthin extraction efficiency and bioavailability can be improved by using nanotechnology to alter microalgal cell architectures and decrease particle size [215].
- Carrier molecules: It has been demonstrated that carotenoids’ intestinal absorption and water dispersibility are increased when they are combined with carrier molecules, such as proteins, phospholipids, or fatty acids. These complexes improve the carotenoids’ solubility and emulsification efficiency, which makes them easier to incorporate micellarly and enhances their bioavailability [216]. According to Focsan et al. (2019), supramolecular complexes of glycyrrhizic acid with β-carotene, canthaxanthin, lutein, zeaxanthin, astaxanthin, and other carotenoids show improved solubility, stability, and bioavailability [217].

Limitation (4): Electing the extraction solvent becomes more complex when bacteria produce xanthophylls and carotenes simultaneously. In addition, the liability to oxidation during the extraction process and the presence of moisture make the downstream process problematic [84]. Choosing the best solvent system and preventing oxidation will be helpful, as mentioned below;

- Lyophilization and supercritical carbon dioxide: As carotenoids are lipophilic in nature, the presence of water in the microbial biomass hinders the extraction process, minimizing the final yield. To prevent the deleterious action of moisture in the extraction procedures, lyophilization of microbial biomass to remove water content and improve the extraction of carotenoids will be effective [155]. As well as using supercritical carbon dioxide is helpful to improve the extraction, for example, using supercritical carbon dioxide as a green solvent to dry the *E. coli* wet biomass and extract lycopene led to 99% successful recovery of lycopene [218].
- Nonrandom two-liquid segment activity coefficient (NRTL-SAC) model: Using the NRTL-SAC model to quickly identify the best solvent solution is advantageous. Initially, the NRTL-SAC model can estimate partition coefficients, which aid in the identification of potential solvent systems. These predictions can be confirmed using experimental techniques, such as thin-layer chromatography or shake-flask testing using a variety of solvent compositions [219].
- Antioxidant: Ascorbic acid, butyl-hydroxytoluene, butyl-hydroquinone, and butyl-hydroxyanisole have been recommended as antioxidants to stabilize carotenoids throughout the extraction process [84].

## Conclusion

This article reviews the bioproduction and biological applications of the fundamental carotenoid pigments, including lycopene, β-carotene, β-cryptoxanthin, zeaxanthin, canthaxanthin, and astaxanthin. First, the current review discussed that bacteria are superior cell factories for natural carotenoid production, more better than other natural sources, having many privileges, such as versatile productivity, production of rare carotenoids, high growth rates, low nutritional demands, easy genetic manipulation, fast extraction, high stability, and low cost. The biosynthesis of these fundamental carotenoids through MVA and MEP pathways was also reviewed. Next, the upstream process steps of bacterial carotenoids were discussed, such as screening of carotenoid-producing bacteria, strategies for improving carotenoid production through optimization of physicochemical parameters via OFAT and RSM strategies, and through metabolic engineering, such as overexpression of key genes or gene knockout in carotenogenic bacteria, and heterogeneous expression of carotenoid genes in non-carotenogenic bacteria. Moreover, the fermentation strategies were detailed in this review, along with carotenoid extraction using organic solvents or other greener solvents, including supercritical fluid extraction, ultrasonic-assisted extraction, microwave-assisted extraction, enzymatic extraction, natural solvent mixtures, ionic liquids, deep eutectic solvents, and the microemulsion technique. After that, the biological activities of these carotenoids were uncovered, such as antibacterial, antibiofilm, antifungal, anticancer, antioxidant, anti-inflammatory, antidiabetic, and food additives. Finally, the review uncovered the limitations of bioproduction of bacterial carotenoids, including high production costs, low yield of rare carotenoids, low stability and bioavailability, and extraction difficulties. As well as the possible tackles of these limitations were discussed, for instance, screening of highly productive strains, co-culturing, low-cost substrate, molecular chaperon, microfluidics, CRISPR, self-assembled protein nanocages, SA-Clo, AI-driven enzyme, emulsion formation, microencapsulation and liposome, nanotechnology, carrier molecules, lyophilization and supercritical carbon dioxide, NRTL-SAC model, and antioxidant.

## Data Availability

All data generated or analyzed during this study are included in this published article.
